# Prevalence of Extremely Severe Obesity and Metabolic Dysfunction Among US Children and Adolescents

**DOI:** 10.1001/jamanetworkopen.2025.21170

**Published:** 2025-07-16

**Authors:** Eliane Münte, Xinlian Zhang, Amit Khurana, Phillipp Hartmann

**Affiliations:** 1 Department of Pediatrics, University of California San Diego, La Jolla; 2 Division of Biostatistics and Bioinformatics, Herbert Wertheim School of Public Health and Human Longevity Science, University of California San Diego, La Jolla; 3 Division of Gastroenterology, Hepatology & Nutrition, Rady Children’s Hospital San Diego, San Diego, California

## Abstract

**Importance:**

Pediatric obesity is associated with complications such as metabolic dysfunction–associated steatotic liver disease (MASLD), type 2 diabetes, dyslipidemia, and hypertension. However, data on the prevalence of extremely severe obesity in childhood and adolescence and its associated complications are lacking.

**Objective:**

To assess trends in pediatric obesity over time and to investigate the association between obesity degree and comorbidities.

**Design, Setting, and Participants:**

This cross-sectional study included data from nationally representative samples of pediatric participants aged 2 to 18 years from the National Health and Nutrition Examination Survey (NHANES) from 2008 through 2023. NHANES is a US cross-sectional survey assessing health and nutrition through questionnaires, physical examinations, and laboratory tests.

**Exposure:**

Pediatric obesity, classified as follows: traditional classes 1 to 3 obesity (body mass index [BMI] ≥95th percentile to <160% of the 95th percentile) and extremely severe obesity, comprising class 4 (BMI ≥160% to <180% of 95th percentile) and class 5 (BMI ≥180% of 95th percentile) obesity.

**Main Outcomes and Measures:**

The main outcomes were national trends in prevalence of obesity classes in childhood and adolescence, stratified by gender, age, and ethnicity and race, and odds of conditions of metabolic dysfunction in each obesity group.

**Results:**

The cohort included 25 847 participants (51% male) with a median age of 10.0 (IQR, 6.00-15.0) years. From 2008 to 2023, the prevalence of pediatric obesity increased, with the highest relative increase of 253.1% observed for extremely severe obesity from 0.32% (95% CI, 0.17%-0.59%) in 2008 to 1.13% (95% CI, 0.78%-1.65%) in 2023. From 2020 to 2023, extremely severe obesity was particularly prevalent among adolescents aged 16 to 18 years (1.99%; 95% CI, 1.31%-2.99%) and non-Hispanic Black participants (2.04%; 95% CI, 1.45%-2.86%). Compared with milder forms of obesity, extremely severe obesity was associated with higher odds of metabolic complications, including MASLD (odds ratio [OR], 6.74; 95% CI, 3.30-15.75), prediabetes or diabetes (OR, 4.94; 95% CI, 3.41-7.14), severe insulin resistance (OR, 8.05; 95% CI, 3.70-17.02), and metabolic syndrome (OR, 1.99; 95% CI, 1.45-2.73).

**Conclusions and Relevance:**

In this nationally representative cross-sectional study of US children and adolescents, prevalence of extremely severe obesity significantly increased over time, particularly among older adolescents and non-Hispanic Black participants. Extremely severe obesity was associated with severe metabolic and cardiovascular complications, including MASLD, prediabetes or diabetes, severe insulin resistance, and metabolic syndrome. These findings emphasize the urgent need for public health interventions and policies to address pediatric obesity broadly.

## Key Points

**Question:**

What is the prevalence of pediatric extremely severe obesity (BMI ≥160% of 95th percentile) in the US, and is it associated with metabolic complications?

**Findings:**

In this nationally representative cross-sectional study of 25 847 children and adolescents, prevalence of extremely severe obesity increased from 0.32% in 2008 to 1.13% in 2023 and was highest among adolescents and non-Hispanic Black individuals. Affected participants had higher risks of metabolic complications, including liver disease, prediabetes and diabetes, severe insulin resistance, and metabolic syndrome, than those with milder or no obesity.

**Meaning:**

The findings suggest that urgent public health interventions are needed to address pediatric obesity.

## Teaser

This cross-sectional study investigates changes in the prevalence of pediatric extremely severe obesity in the US from 2008 through 2023 and the association of pediatric extremely severe obesity with metabolic comorbidities.

## Introduction

The World Health Organization recently reported that in 2022, 37 million children younger than 5 years and over 390 million aged 5 to 19 years had overweight, including 160 million with obesity, underscoring a dramatic rise in pediatric obesity rates, which have quadrupled since 1990.^1,2^ The escalating prevalence and severe health consequences of childhood obesity recently led Bomberg et al^3^ to suggest pediatric obesity as a public health emergency in the US. Childhood obesity is associated with a range of metabolic disorders, including metabolic dysfunction–associated steatotic liver disease (MASLD), which is prevalent in about 38% of children with obesity.^4^ Additionally, type 2 diabetes, metabolic syndrome, and dyslipidemia affect approximately 40% of children with obesity.^5,6,7,8^ Furthermore, about a quarter of children with obesity have hypertension.^9^

While the detrimental health effects of childhood obesity are undisputed, data are still lacking on how obesity severity relates to specific comorbidities in childhood. In the US, pediatric obesity is usually classified as a body mass index (BMI; calculated as weight in kilograms divided by height in meters squared) at or above the 95th percentile, with classes 2 and 3 obesity (severe obesity) defined as 120% to less than 140% and 140% or more of the 95th percentile, respectively.^7^ In this study, we focused on the prevalence and associated comorbidities of extremely severe obesity, referred to as class 4 (BMI ≥160% to <180% of 95th percentile) and class 5 (BMI ≥180% of 95th percentile) obesity.

In this study, data from 25 847 participants of the National Health and Nutrition Examination Survey (NHANES) from 2008 to 2023 were analyzed. We hypothesized that extremely severe pediatric obesity (ie, classes 4 and 5) increased in prevalence over the observation period. Moreover, we hypothesized that markers of metabolic dysfunction would show more severe alterations in obesity classes 4 and 5 compared with lower classes of obesity.

## Methods

### Data Source

NHANES is a nationally representative, cross-sectional survey conducted among US residents every 2 years. Managed by the National Center for Health Statistics and the Centers for Disease Control and Prevention (CDC), NHANES aims to assess the health and nutritional status of the US population. As part of the survey, participants provide demographic information, undergo a physical examination and blood and imaging studies, and complete validated questionnaires about their health behaviors.^10^ For the present cross-sectional study, we obtained the NHANES datasets for participants aged 2 to 18 years for 2007-2008, 2009-2010, 2011-2012, 2013-2014, 2015-2016, 2017-2018, 2017-2020, and 2021-2023 (hereafter, NHANES 2008, 2010, 2012, 2014, 2016, 2018, 2020, and 2023, respectively) using the nhanesA library, version 1.3, in R, version 4.4.2 (R Project for Statistical Computing).^11^ This study was exempt from institutional review board approval and informed consent because it used publicly available, deidentified data from the NHANES, which is overseen by the National Center for Health Statistics research ethics review board, a part of the CDC.^10^ The study followed the Strengthening the Reporting of Observational Studies in Epidemiology (STROBE) reporting guideline for cross-sectional studies.

Ethnicity and race, ascertained by self-report during the NHANES household interview, were included in our analysis to examine potential disparities in health outcomes and to control for sociodemographic factors that may influence the variables of interest. Categories were those included in the NHANES: Mexican American, non-Hispanic Black, non-Hispanic White, Other Hispanic (included respondents who self-identified as Hispanic but not as Mexican American), and multiracial or other race (included non-Hispanic individuals who identified as races other than Black or White).

### Definition of Different Classes of Pediatric Obesity

We defined different obesity classes based on the 95th percentile of the BMI per age and gender, as typical in pediatrics,^7^ with the addition of classes 4 and 5: class 1 (BMI ≥95th percentile to <120% of 95th percentile), class 2 (BMI ≥120% to <140% of 95th percentile), class 3 (BMI ≥140% to <160% of 95th percentile), class 4 (BMI ≥160% to <180% of 95th percentile), and class 5 (BMI ≥180% of 95th percentile). Pediatric obesity classes 4 and 5 correspond to adult obesity classes 4 and 5^12^ (eTable 1 in Supplement 1). Updated age- and gender-specific BMI percentiles and extended BMI percentiles were calculated using the cdcanthro library, version 0.1.3, in R.^13^

### Definitions of Conditions of Metabolic Dysfunction

MASLD was defined according to Rinella et al.^14^ Liver imaging was performed using FibroScan for controlled attenuation parameter (CAP), an indirect marker for hepatic steatosis, and for liver stiffness, a marker for hepatic fibrosis. After ruling out hepatitis B and C, autoimmune hepatitis, and other liver diseases, based on questionnaires and serologic test results as available, MASLD was defined by presence of hepatic steatosis and at least 1 of 5 cardiometabolic criteria: overweight or obesity per BMI at or above the 85th percentile for age and gender or waist circumference above the 95th percentile; prediabetes or diabetes per glycated hemoglobin (HbA_1c_) level of 5.7% of total hemoglobin or greater (to convert to proportion of total hemoglobin, multiply by 0.01), fasting serum glucose level of 100 mg/dL or greater (to convert to mmol/L, multiply by 0.0555), or prior diagnosis of prediabetes or diabetes; arterial hypertension per blood pressure of 130/85 mm Hg or greater for age 13 years or older and systolic and/or diastolic blood pressure at or above the 95th percentile or blood pressure of 130/80 mm Hg or greater for those younger than 13 years or receipt of antihypertensive treatment; hypertriglyceridemia per fasting triglyceride levels of 150 mg/dL or greater for age 10 years or older and 100 mg/dL or greater for those younger than 10 years (to convert to mmol/L, multiply by 0.0113) or receipt of lipid-lowering treatment; or high-density lipoprotein (HDL) level of 40 mg/dL or lower (to convert to mmol/L, multiply by 0.0259).^14^ The childsds library, version 0.9.8, in R was used to calculate the waist circumference percentiles based on waist circumference, age, and gender using the us.ref reference.^15^ Systolic and diastolic blood pressure percentiles were calculated using the pedbp library, version 2.0.2, in R.^16^ Hepatic steatosis was defined by transient elastography using a cutoff of 277 dB/m per CAP.^17^ Hepatic steatosis grades 1 or higher, 2 or higher, and 3 were defined by a CAP of 277 dB/m or greater, 294 dB/m or greater, and 349 dB/m or greater, respectively, as previously described.^17^ Hepatic fibrosis stage 2 or higher and 3 or higher were defined using cutoffs of 7.4 kPa and 10.2 kPa, respectively, as previously described by Nobili et al.^18^ Transient elastography findings were included in the 2018, 2020, and 2023 NHANES datasets.

Following the definition by de Ferranti et al,^19^ we diagnosed pediatric metabolic syndrome if at least 3 of 5 cardiometabolic criteria were met: waist circumference above the 75th percentile for age and gender, fasting serum glucose level of 110 mg/dL or greater, fasting triglycerides level of 100 mg/dL or greater, HDL cholesterol level of 50 mg/dL or less, or systolic and/or diastolic blood pressure above the 90th percentile for age, gender, and height.^19,20^ Missing data on continuous cardiometabolic criteria for metabolic syndrome prevalence were imputed using multivariate imputation via chained equations following the classification and regression tree algorithm using the mice library, version 3.17.0, in R.^21,22^ Imputation of all missing variables was performed simultaneously and based on cardiometabolic parameters, including waist circumference and HbA_1c_, fasting glucose, insulin, triglycerides, and HDL levels as well as systolic and diastolic blood pressure. We imputed 5 complete datasets as recommended.^23^

For the purposes of this study, the prevalence of prediabetes or diabetes was determined by an HbA_1c_ level of 5.7% of total hemoglobin and history of prediabetes or diabetes. The Homeostatic Model Assessment for Insulin Resistance (HOMA-IR) is calculated as (fasting insulin [μIU/mL] × fasting glucose [mg/dL])/405.^24^ Pediatric insulin resistance was defined as a HOMA-IR of 3.16 or greater.^25^ Severe insulin resistance was defined by a fasting insulin level of 50 μIU/mL or greater (to convert to pmol/L, multiply by 6.945).^26^

### Statistical Analysis

Results are presented as median (IQR) unless specified otherwise. Comparisons between 2 groups for continuous variables were made using the Wilcoxon Mann-Whitney rank sum test. For comparisons among 3 or more groups, the Kruskal-Wallis test was used. If the Kruskal-Wallis test indicated statistical significance, pairwise Wilcoxon Mann-Whitney rank sum tests with Holm correction were conducted, and the significant adjusted *P* values were reported in the figures. Comparisons between 2 or more groups for prevalence data were made using the Pearson χ^2^ test, and for more than 2 groups, pairwise comparisons with Holm corrections were performed. All statistical tests were 2-sided. Pearson correlation analyses were conducted to examine correlations between variables, with *P* values adjusted for multiplicity using Holm correction where indicated. Multiple logistic regression and odds ratio (OR) calculations were carried out using the stats, version 4.4.2, and questionr, version 0.7.8, libraries in R, respectively. Two-sided *P* < .05 was considered statistically significant for all tests. Statistical analyses were performed using R, version 4.4.2, and RStudio, version 2024.12.0 + 467 for Mac, 2020. Additional methods are described in the eMethods in Supplement 1.

## Results

### Demographics and Clinical Data

The combined 2008-2023 NHANES pediatric cohort consisted of 25 847 participants. The Table summarizes a wide range of demographic, physical examination, laboratory, and imaging data obtained for the cohort. The gender distribution was almost evenly split between female (49.0%) and male (51.0%) participants. The median age was 10.0 years (IQR, 6.00-15.0 years). Regarding ethnicity and race, 20.3% of participants were Mexican American, 23.9% were non-Hispanic Black, 30.0% were non-Hispanic White, 11.1% were Other Hispanic, and 14.6% were from other racial backgrounds, including multiracial. The median BMI was 18.4 (IQR, 16.1-22.6). Key metabolic parameters included HbA_1c_, fasting glucose, fasting insulin, HOMA-IR, and the lipid profile. In addition, results of liver imaging for CAP and liver stiffness were included. Detailed values are provided in the Table.

**Table.** Demographic, Physical Examination, Laboratory, and Imaging Data for the Study Population From NHANES Datasets From 2008 to 2023

Characteristic	Participants^a^
Gender, No. (%) (N = 25 847)	
Female	12 672 (49.0)
Male	13 175 (51.0)
Age, y (N = 25 847)	10.0 (6.00-15.0)
Ethnicity and race, No. (%) (N = 25 847)^b^	
Mexican American	5242 (20.3)
Non-Hispanic Black	6186 (23.9)
Non-Hispanic White	7761 (30.0)
Other Hispanic	2874 (11.1)
Multiracial or other race	3784 (14.6)
Height, cm (N = 25 847)	139 (115-160)
Body weight, kg (N = 25 847)	35.9 (21.2-57.3)
BMI (N = 25 847)	18.4 (16.1-22.6)
Waist circumference, cm (n = 25 649)	65.2 (54.3-77.7)
Pulse, bpm (n = 14 414)	78.0 (70.0-86.0)
Blood pressure, mm Hg (n = 13 935)	
Systolic	105.3 (98.7-112.0)
Diastolic	59.3 (52.7-66.0)
White blood cell count, /μL (n = 20 741)	6900 (5700-8400)
Hemoglobin, g/dL (n = 20 742)	13.1 (12.4-13.9)
Hematocrit, % (n = 20 742)	38.6 (36.7-41.0)
Platelets, ×10^3^/μL (n = 20 742)	282 (242-328)
C-reactive protein, mg/dL (n = 9437)	0.048 (0.025-0.131)
Sodium, mEq/L (n = 7185)	140 (138-141)
Potassium, mEq/L (n = 7183)	4.00 (3.81-4.20)
Chloride, mEq/L (n = 7184)	104 (102-105)
Bicarbonate, mEq/L (n = 7185)	25.0 (23.0-26.0)
Blood urea nitrogen, mg/dL (n = 7185)	11.0 (9.0-13.0)
Creatinine, mg/dL (n = 7185)	0.69 (0.59-0.82)
Alanine aminotransferase, U/L (n = 7185)	15.0 (12.0-20.0)
Aspartate aminotransferase, U/L (n = 7179)	21.0 (18.0-25.0)
Alkaline phosphatase, U/L (n = 7187)	109.0 (76.0-192.0)
Total bilirubin, mg/dL (n = 7185)	0.60 (0.40-0.80)
Albumin, g/dL (n = 7187)	4.40 (4.20-4.60)
Total protein, g/dL (n = 7184)	7.20 (7.00-7.50)
γ-Glutamyltransferase, U/L (n = 7185)	13.0 (10.0-17.0)
HbA_1c_, % of total hemoglobin (n = 8204)	5.30 (5.10-5.50)
Fasting serum glucose, mg/dL (n = 3854)	95.0 (90.0-100.0)
Fasting insulin, μIU/mL (n = 3716)	11.5 (7.7-17.7)
HOMA-IR (n = 3707)^c^	2.67 (1.77-4.17)
Fasting triglycerides, mg/dL (n = 3348)	63.0 (45.0-90.0)
Fasting cholesterol, mg/dL	
Total (n = 15 573)	155.0 (138.0-174.0)
LDL (n = 3343)	84.0 (69.0-102.0)
HDL (n = 15 574)	52.0 (45.0-61.0)
Median CAP, dB/m (n = 3383)	215 (184-252)
Median liver stiffness, kPa (n = 3485)	4.70 (4.00-5.70)

Abbreviations: bpm, beats per minute; BMI, body mass index (calculated as weight in kilograms divided by height in meters squared); CAP, controlled attenuation parameter; HbA_1c_, glycated hemoglobin; HDL, high-density lipoprotein; HOMA-IR, Homeostatic Model Assessment of Insulin Resistance; LDL, low-density lipoprotein; NHANES, National Health and Nutrition Examination Survey.

SI conversion factors: To convert alanine aminotransferase to μkat/L, multiply by 0.0167; albumin to g/L, multiply by 10; alkaline phosphatase to μkat/L, multiply by 0.0167; aspartate aminotransferase to μkat/L, multiply by 0.0167; bicarbonate to mmol/L, multiply by 1.0; chloride to mmol/L, multiply by 1.0; C-reactive protein to mg/L, multiply by 10; creatinine to μmol/L, multiply by 88.4; γ-glutamyltransferase to μkat/L, multiply by 0.0167; HbA_1c_ to proportion of total hemoglobin, multiply by 0.01; HDL to mmol/L, multiply by 0.0259; hematocrit to proportion of 1.0, multiply by 0.01; hemoglobin to g/L, multiply by 10.0; insulin to pmol/L, multiply by 6.945; LDL to mmol/L, multiply by 0.0259; platelets to ×10^9^/L, multiply by 1.0; potassium to mmol/L, multiply by 1.0; protein to g/L, multiply by 10.0; serum glucose to mmol/L, multiply by 0.0555; sodium to mmol/L, multiply by 1.0; total bilirubin to μmol/L, multiply by 17.104; total cholesterol to mmol/L, multiply by 0.0259; triglycerides to mmol/L, multiply by 0.0113; white blood cell count to ×10^9^/L, multiply by 0.001.

^a^ Data are presented as median (IQR) unless otherwise indicated.

^b^ Other Hispanic includes respondents who self-identified as Hispanic but not as Mexican American. Other race includes non-Hispanic individuals who identified as races other than Black or White and multiracial respondents.

^c^ HOMA-IR is calculated as (fasting insulin [μIU/mL] × fasting glucose [mg/dL])/405.

### Prevalence of Extremely Severe Pediatric Obesity

**Figure 1.**
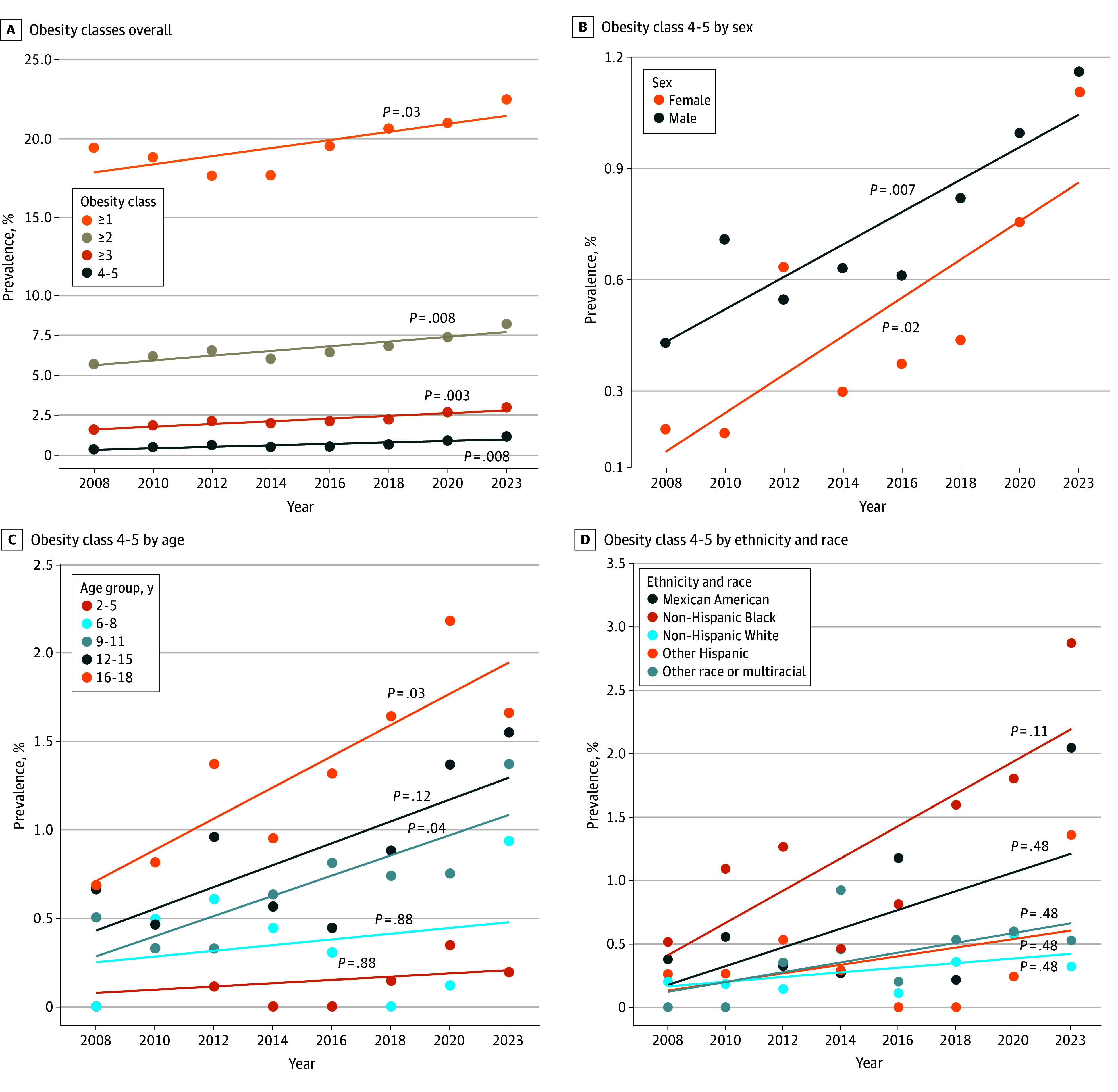
Prevalence of Pediatric Obesity From 2008 to 2023 Among 25 847 Individuals Adjusted *P* values after Holm correction are shown.

The prevalence of any obesity increased from 19.46% (95% CI, 18.11%-20.89%) in 2008 to 22.52% (95% CI, 20.89%-24.24%) (*P* = .02) in 2023; obesity class 2 or higher, from 5.73% (95% CI, 4.96%-6.60%) in 2008 to 8.28% (95% CI, 7.24%-9.45%) (*P* = .001) in 2023; obesity class 3 or higher, from 1.58% (95% CI, 1.19%-2.08%) in 2008 to 2.98% (95% CI, 2.37%-3.75%) (*P* = .002) in 2023; and obesity classes 4 to 5, from 0.32% (95% CI, 0.17%-0.59%) in 2008 to 1.13% (95% CI, 0.78%-1.65%) (*P* = .001) in 2023. These increases corresponded to relative increases of 15.7% for any obesity, 44.5% for obesity class 2 or higher, 88.6% for obesity class 3 or higher, and 253.1% for obesity class 4 to 5 during that period. The increases in prevalence were consistent throughout that period, particularly for classes 2 or higher (*R*, 0.90; *P* = .008), 3 or higher (*R*, 0.94; *P* = .003), and 4 to 5 (*R*, 0.88; *P* = .008) (Figure 1A). Similarly, a positive linear correlation existed for the prevalence of classes 4 to 5 obesity for males (*R*, 0.88; *P* = .007) and females (*R*, 0.80; *P* = .02) (Figure 1B). The prevalence of extremely severe obesity (classes 4-5) increased steadily, especially among adolescents aged 16 to 18 years (*R*, 0.86; *P* = .03), with 1.99% (95% CI, 1.31%-2.99%) fulfilling the criteria in 2020-2023; this prevalence was significantly higher than the 0.30% (95% CI, 0.13%-0.70%) among children aged 2 to 5 years and 0.39% (95% CI, 0.17%-0.92%) among those aged 6 to 8 years (Figure 1C and eFigure 1 in Supplement 1).

No significant correlations were found when stratified by ethnicity and race between 2008 and 2023 (Figure 1D). However, non-Hispanic Black individuals had the highest prevalence of classes 4 to 5 obesity in 2020-2023 (2.04%; 95% CI, 1.45%-2.86%), markedly higher than the prevalence among non-Hispanic White individuals (0.47%; 95% CI, 0.26%-0.84%) (*P* < .001) and those who reported other races or multiracial (0.57%; 95% CI, 0.28%-1.18%) (*P* = .01) (eFigure 1 in Supplement 1). To identify factors associated with obesity classes 4 to 5, we performed multiple logistic regression using demographic parameters and year of the NHANES dataset iteration in 25 847 participants (eTable 2 in Supplement 1). Factors with significant associations included non-Hispanic Black and Mexican American ethnicity and race (compared with non-Hispanic White), male gender, age, and NHANES dataset iteration (eTable 2 in Supplement 1). The OR for presence of obesity classes 4 to 5 for non-Hispanic Black individuals (compared with all other race and ethnicity categories combined) was 2.89 (95% CI, 2.11-3.96), for males compared with females was 1.50 (95% CI, 1.09-2.07), for ages 12 years or older vs younger than 12 years was 3.19 (95% CI, 2.29-4.52), and for the combined 2020 and 2023 NHANES datasets vs 2008 through 2018 was 1.97 (95% CI, 1.43-2.70) (eTable 3 in Supplement 1).

### Association of Extremely Severe Pediatric Obesity With MASLD and Advanced Fibrosis

**Figure 2.**
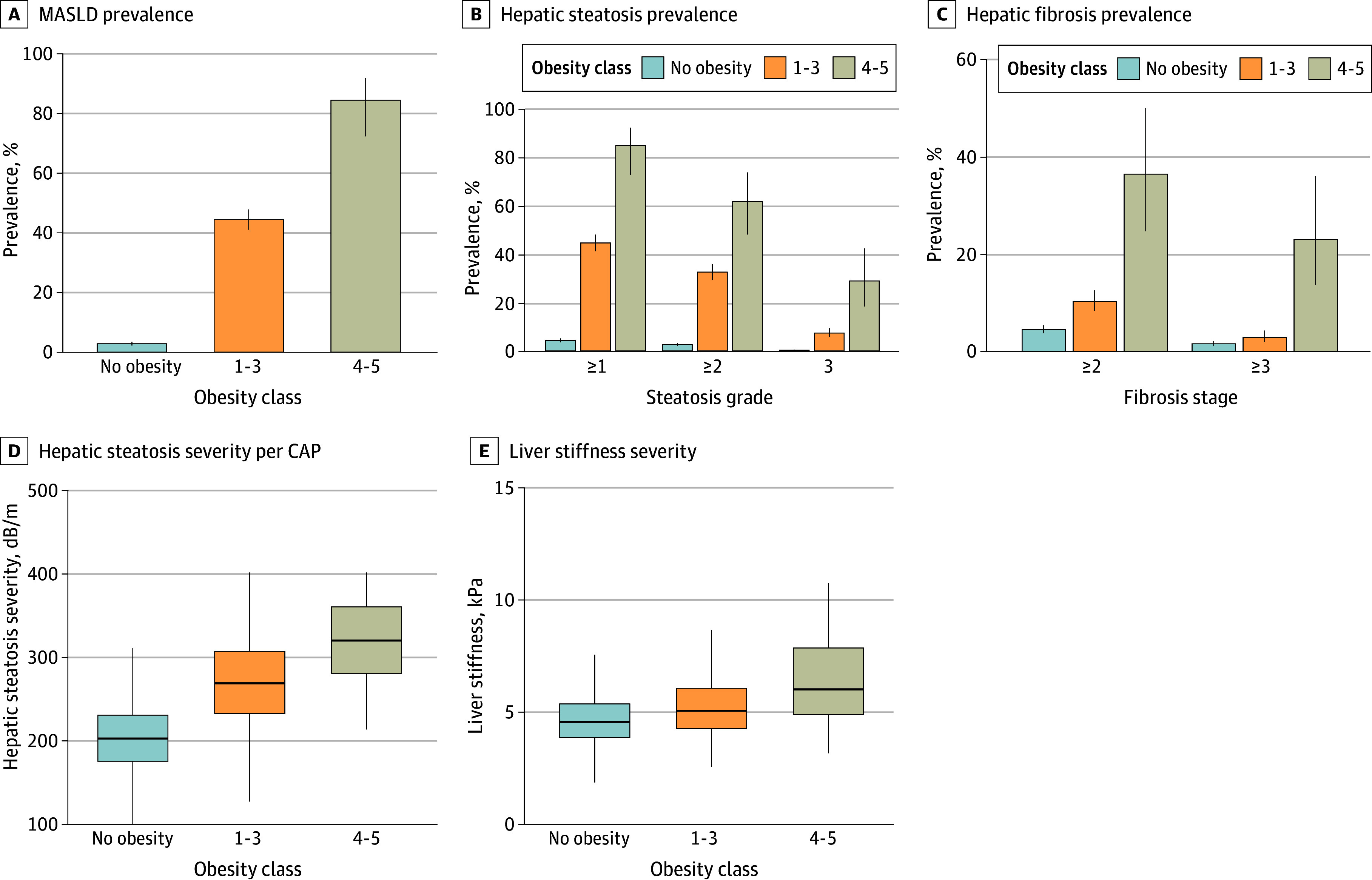
Metabolic Dysfunction-Associated Steatotic Liver Disease (MASLD) and Advanced Liver Fibrosis in Children With Extremely Severe Obesity Adjusted *P* < .001 for all comparisons after Holm correction in A, B, D, and E and for fibrosis stage 2 or higher in C. A-C, Whiskers indicate 95% CIs. D and E, The box extends from the 25th to 75th percentile, with the center line indicating the median; the bottom whiskers indicate the minimum value of the data that is within 1.5 times the IQR under the 25th percentile, and the top whiskers indicate the maximum value of the data that is within 1.5 times the IQR over the 75th percentile. CAP indicates controlled attenuation parameter.

Since obesity is associated with MASLD,^27,28,29^ we evaluated whether pediatric extremely severe obesity is also associated with a higher risk of MASLD and with higher severity of MASLD compared with lower classes of obesity. After ruling out other steatogenic causes, the prevalence of MASLD was the highest among individuals with obesity classes 4 to 5 (84.62%; 95% CI, 72.48%-91.99%) compared with obesity classes 1 to 3 (44.48%; 95% CI, 41.13%-47.89%) and no obesity (2.80%; 95% CI, 2.23%-3.51%) (*P* < .001 for all comparisons after adjustment) and was significantly higher compared with class 3 separately (Figure 2A and eFigure 2 in Supplement 1). The OR for MASLD in obesity classes 4 to 5 vs 1 to 3 was 6.74 (95% CI, 3.30-15.75) (eTable 4 in Supplement 1).

Likewise, the prevalence of steatosis grade 1 or higher (≥277 dB/m), 2 or higher (≥294 dB/m), and 3 (≥349 dB/m) increased with higher obesity class, with significant differences between individuals without obesity (grade ≥1, 4.30% [95% CI, 3.58%-5.15%]; grade ≥2, 2.61% [95% CI, 2.06%-3.29%]; grade 3, 0.27% [95% CI, 0.13%-0.55%]), those with obesity classes 1 to 3 (grade ≥1, 44.48% [95% CI, 41.13%-47.89%]; grade ≥2, 32.48% [95% CI, 29.38%-35.76%]; grade 3, 7.39% [95% CI, 5.80%-9.38%]), and those with obesity classes 4 to 5 (grade ≥1, 84.62% [95% CI, 72.48%-91.99%]; grade ≥2, 61.54% [95% CI, 47.96%-73.53%]; grade 3, 28.85% [95% CI, 18.33%-42.27%]) (Figure 2B and eFigure 2 in Supplement 1). This also held true for the prevalence of fibrosis stage 2 or higher (>7.4 kPa) and 3 or higher (>10.2 kPa). Participants with extremely severe obesity had a significantly higher prevalence of fibrosis stages 2 or higher (36.54%; 95% CI, 24.80%-50.13%) and 3 or higher (23.08%; 95% CI, 13.72%-36.13%) than participants with classes 1 to 3 obesity (stage ≥2, 10.30% [95% CI, 8.41%-12.56%]; stage ≥3, 2.91% [95% CI, 1.96%-4.29%]); the prevalence of advanced fibrosis was significantly higher among participants with obesity classes 1 to 3 than among participants without obesity (stage ≥2, 4.53% [95% CI, 3.79%-5.40%]; stage ≥3, 1.57% [95% CI, 1.16%-2.13%]) (Figure 2C and eFigure 2 in Supplement 1). The OR for advanced fibrosis associated with obesity classes 4 to 5 compared with classes 1 to 3 was 10.00 (95% CI, 4.51-21.24) (eTable 4 in Supplement 1).

The median value for hepatic steatosis was markedly higher in individuals with obesity classes 4 to 5 (319 dB/m [IQR, 280-359 dB/m]) than in those with classes 1 to 3 (268 dB/m [IQR, 232-306 dB/m]) and in individuals without obesity (202 dB/m [IQR, 175-230 dB/m]) (Figure 2D). Furthermore, median liver stiffness values reached 6.4 kPa (IQR, 5.2-8.6 kPa) in individuals with obesity classes 4 to 5, which was significantly higher than the 5.1 kPa (IQR, 4.3-6.1 kPa) observed in individuals with obesity classes 1 to 3 and 4.6 kPa (IQR, 3.9-5.5 kPa) in individuals without obesity (Figure 2E). Of note, the alanine aminotransferase and γ-glutamyltransferase levels were significantly higher in individuals with extremely severe obesity compared with those with milder forms of obesity and those with no obesity (eFigure 3 in Supplement 1).

### Association of Extremely Severe Pediatric Obesity With Prediabetes, Diabetes, and Severe Insulin Resistance

**Figure 3.**
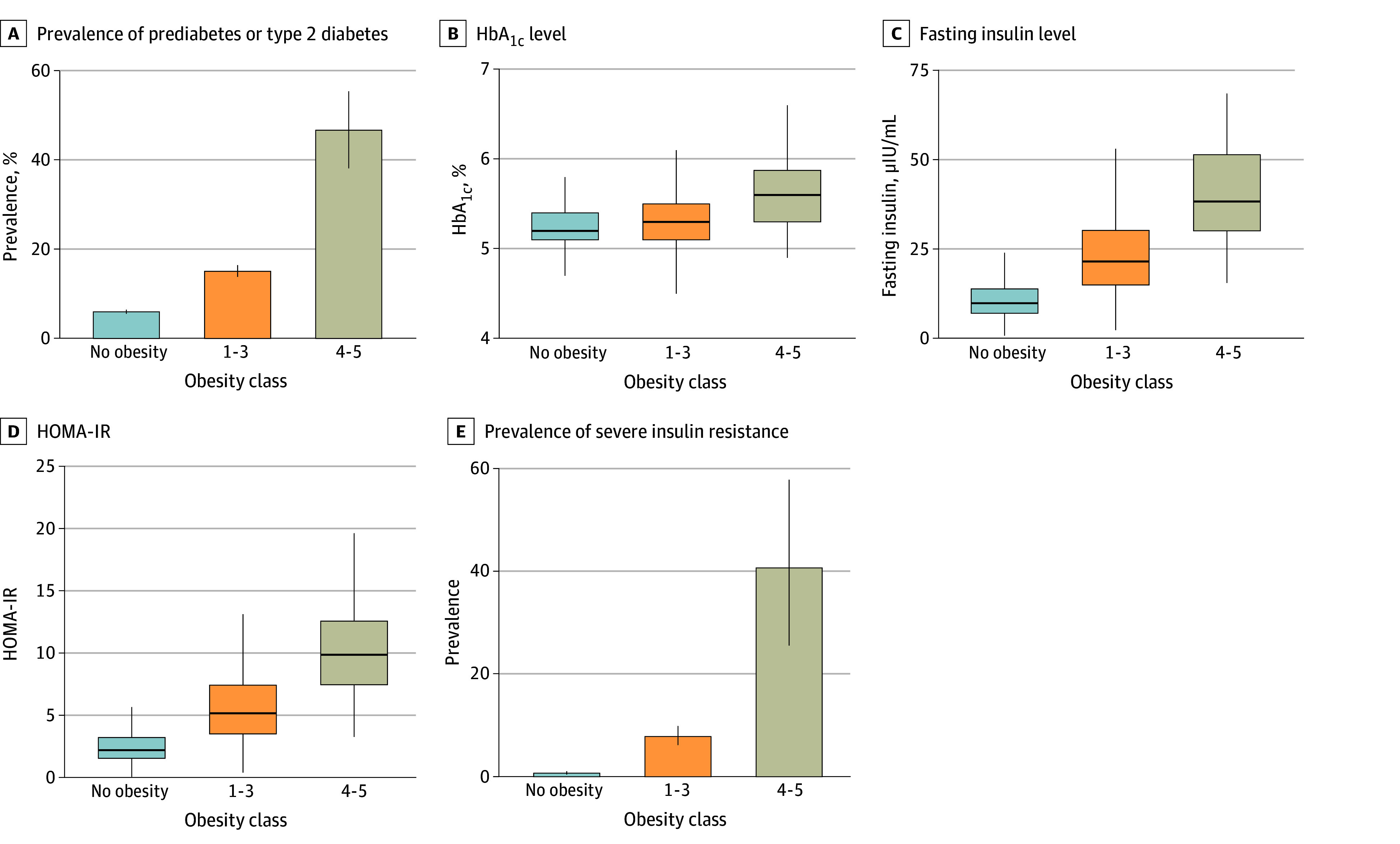
Prediabetes, Diabetes, and Severe Insulin Resistance in Children With Extremely Severe Obesity Adjusted *P* < .001 for all comparisons after Holm correction. A, E, Whiskers indicate 95% CIs. B-D, The box extends from the 25th to 75th percentile, with the center line indicating the median; the bottom whiskers indicate the minimum value of the data that is within 1.5 times the IQR under the 25th percentile, and the top whiskers indicate the maximum value of the data that is within 1.5 times the IQR over the 75th percentile. HbA_1c_ indicates glycated hemoglobin; HOMA-IR, Homeostatic Model Assessment of Insulin Resistance (calculated as [fasting insulin (μIU/mL) × fasting glucose (mg/dL)]/405).

Next, since obesity is associated with increased risk of diabetes and insulin resistance,^30,31^ we investigated whether these conditions were exacerbated in individuals with obesity classes 4 to 5 compared with groups with lower BMI percentiles. The prevalence of prediabetes or type 2 diabetes was significantly higher among participants with obesity classes 4 to 5 (46.77%; 95% CI, 38.22%-55.52%) than among those with classes 1 to 3 (15.10%; 95% CI, 13.83%-16.46%) and those without obesity (5.99%; 95% CI, 5.56%-6.46%) (*P* < .001 for all comparisons after adjustment) (Figure 3A). The prevalence was also higher when obesity classes 4 to 5 were compared with class 3 alone (eFigure 4 in Supplement 1). The OR for prediabetes or type 2 diabetes associated with obesity classes 4 to 5 compared with classes 1 to 3 was 4.94 (95% CI, 3.41-7.14) (eTable 4 in Supplement 1). The median HbA_1c_ level was significantly higher in individuals with classes 4 to 5 obesity (5.6% [IQR, 5.3%-5.9%]) than in those with classes 1 to 3 obesity (5.3% [IQR, 5.1%-5.3%]) or no obesity (5.2% [IQR, 5.1%-5.4%]) (Figure 3B and eFigure 4 in Supplement 1). For fasting glucose, the median value was significantly higher in individuals with obesity classes 1 to 3 compared with those without obesity. In contrast, individuals with obesity classes 4 to 5 did not have a significant difference in fasting glucose level compared with those with classes 1 to 3 or no obesity. Individuals with obesity classes 4 to 5 had significantly higher median fasting insulin levels (43.42 μIU/mL [IQR, 32.32-61.02 μIU/mL]) compared with those with obesity classes 1 to 3 (21.75 μIU/mL [IQR, 15.05-30.78 μIU/mL]) and those without obesity (9.77 μIU/mL [IQR 6.97-13.85 μIU/mL]) (Figure 3C and eFigure 5 in Supplement 1).

Similarly, the HOMA-IR was more elevated in individuals with obesity classes 4 to 5 (median, 10.15 [IQR, 8.14-15.17]) than in those with classes 1 to 3 (5.25 [IQR, 3.58-7.55]) or no obesity (2.27 [IQR, 1.61-3.28]) (all *P* < .001) (Figure 3D and eFigure 5 in Supplement 1). It has been suggested that the pediatric population, in particular the adolescent population, may have higher insulin resistance than adults.^32,33^ A higher HOMA-IR cutoff of 3.16 was hence suggested to define insulin resistance in the pediatric population compared with 2.5 in adults.^25^ However, even with the higher cutoff, 100% (95% CI, 89.28%-100%) of participants with obesity classes 4 to 5 met the definition of insulin resistance compared with 80.88% (95% CI, 78.04%-83.43%) of those with obesity classes 1 to 3 (*P* = .006) and 27.49% (95% CI, 25.89%-29.16%) of those without obesity (*P* < .001 for comparisons with either group with obesity). In addition, severe insulin resistance, defined by a fasting insulin level of 50 μIU/mL or greater,^26^ was significantly more prevalent in individuals with obesity classes 4 to 5 (40.63%; 95% CI, 25.52%-57.74%) than in those with obesity classes 1 to 3 (7.82%; 95% CI, 6.17%-9.87%) or no obesity (0.70%; 95% CI, 0.45%-1.08%) and was also significantly higher compared with class 3 separately (Figure 3E and eFigure 5 in Supplement 1). The OR for severe insulin resistance associated with obesity classes 4 to 5 compared with classes 1 to 3 was 8.05 (95% CI, 3.70-17.02) (eTable 4 in Supplement 1).

### Association of Extremely Severe Pediatric Obesity With Other Cardiometabolic Risk Factors

**Figure 4.**
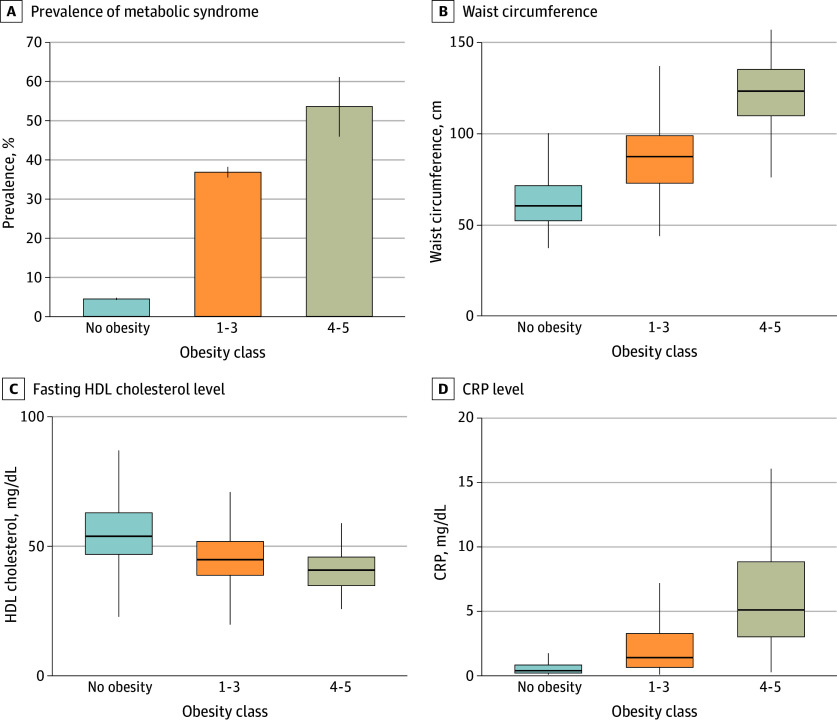
Other Cardiometabolic Risk Factors, Including Metabolic Syndrome, in Children With Extremely Severe Obesity Adjusted *P* < .001 for all comparisons after Holm correction. A, Whiskers indicate 95% CIs. B-D, The box extends from the 25th to 75th percentile, with the center line indicating the median; the bottom whiskers indicate the minimum value of the data that is within 1.5 times the IQR under the 25th percentile, and the top whiskers indicate the maximum value of the data that is within 1.5 times the IQR over the 75th percentile. To convert C-reactive protein (CRP) to mg/L, multiply by 10; high-density lipoprotein (HDL) cholesterol to mmol/L, multiply by 0.0259.

Obesity is associated with cardiometabolic risk factors, including metabolic syndrome.^34,35^ We therefore evaluated whether extremely severe pediatric obesity was associated with additional cardiometabolic risk compared with less severe and no obesity. The prevalence of pediatric metabolic syndrome (at least 3 of the following: increased waist circumference, glucose intolerance, hypertriglyceridemia, low HDL cholesterol level, or elevated blood pressure) was 4.44% (95% CI, 4.17%-4.73%) for participants without obesity, 36.91% (95% CI, 35.58%-38.27%) for those with obesity classes 1 to 3, and 53.75% (95% CI, 46.03%-61.30%) for those with obesity classes 4 to 5 (*P* < .001 for all comparisons after adjustment) (Figure 4A). The prevalence of metabolic syndrome among individuals with obesity classes 4 to 5 was similar to that among individuals with class 3 (eFigure 6 in Supplement 1). The OR for the association of metabolic syndrome with obesity classes 4 to 5 vs classes 1 to 3 was 1.99 (95% CI, 1.45-2.73) (eTable 4 in Supplement 1).

The median waist circumference was 61.0 cm (IQR, 52.8-72.1 cm) in the group without obesity, 88.1 cm (IQR, 73.5-99.6 cm) in the group with obesity classes 1 to 3, and 124.2 cm (IQR, 110.6-136.1 cm) in the group with obesity classes 4 to 5 (*P* < .001 for all comparisons) (Figure 4B and eFigure 6 in Supplement 1). Similarly, the waist circumference percentiles were significantly higher in those with obesity classes 4 to 5 than in those with classes 1 to 3 and in those with classes 1 to 3 than in those without obesity. Patients meeting the criteria for obesity classes 4 to 5 had significantly lower median HDL cholesterol levels (41 mg/dL [IQR, 35-46 mg/dL]) compared with patients with classes 1 to 3 (45 mg/dL [IQR, 39-52 mg/dL]) or no obesity (54 mg/dL [IQR, 47-63 mg/dL]) (Figure 4C and eFigure 6 in Supplement 1). Of note, triglyceride levels were not significantly different between participants with extremely severe obesity and milder forms of obesity (eFigure 6 in Supplement 1). Systolic and diastolic blood pressure were significantly different between all groups and highest in participants with obesity classes 4 to 5 (eFigure 7 in Supplement 1). Systemic inflammation, as measured by serum C-reactive protein level, was significantly more severe in obesity classes 4 to 5 than in obesity classes 1 to 3 and no obesity (Figure 4D and eFigure 7 in Supplement 1).

Of note, when associations of obesity classes with metabolic diseases were stratified by demographic characteristics, males had a higher prevalence of metabolic diseases than females in most comparisons (eFigure 8 in Supplement 1) and children had a lower prevalence of metabolic syndrome than did adolescents (eFigure 9 in Supplement 1). Mexican American participants had a high prevalence of MASLD and severe insulin resistance, while non-Hispanic Black participants had a low prevalence of MASLD and metabolic syndrome. Non-Hispanic Black participants had a higher prevalence of prediabetes or diabetes compared with participants of other ethnicities and races, specifically when participants with obesity were being compared (eFigure 10 in Supplement 1).

## Discussion

Based on NHANES data from 25 847 participants, our study found an increase in prevalence of extremely severe pediatric obesity from 0.32% to 1.13% from 2008 to 2023. We further found that extremely severe pediatric obesity was associated with numerous comorbidities, surpassing those associated with lower obesity classes. Specifically, children with extremely severe obesity had higher risk of MASLD, advanced fibrosis, prediabetes or diabetes, severe insulin resistance, and metabolic syndrome compared with those with obesity classes 1 to 3. The present study has facilitated a better understanding of the health implications associated with extremely severe obesity (BMI ≥160% of the 95th percentile) in children and adolescents in comparison with those without obesity and, in particular, compared with those with obesity classes 1 to 3 (BMI ≥95th percentile to <160% of the 95th percentile).

Our analysis revealed a significant upward trend in the prevalence of pediatric obesity, particularly extremely severe obesity, with a prevalence of 1.13% in 2023 (Figure 1). The increase in prevalence of pediatric obesity is consistent with data from the World Health Organization indicating a quadrupling of pediatric obesity rates from 1990 to 2022 worldwide.^1^ Our study found that male gender was significantly associated with extremely severe obesity, aligning with a recent meta-analysis indicating a 25% higher relative prevalence of obesity in males compared with females in the pediatric population.^36^ Global trends showed a 50% higher prevalence of overweight and obesity in adolescents than in children^36^; our study expanded this to extremely severe obesity, which was particularly prevalent in adolescents. While previous research demonstrated that non-Hispanic Black individuals have a high prevalence of pediatric obesity in general,^37,38^ our study found that non-Hispanic Black participants had a particularly high prevalence of obesity classes 4 to 5, reflecting racial and ethnic disparities in obesity and highlighting the need for targeted health interventions.

This study found an association between higher obesity classes and increased metabolic dysfunction. For instance, participants with obesity classes 4 to 5 had substantially higher rates of MASLD compared with those with lower BMI categories (Figure 2). This finding was consistent across multiple measures of liver health, reinforcing the critical association of extremely severe obesity with liver function and adding to previous investigations of the association between MASLD and the degree of obesity.^4,28,29^ Additionally, liver steatosis may progress to metabolic dysfunction–associated steatohepatitis and liver fibrosis, potentially leading to cirrhosis and hepatocellular carcinoma over time and resulting in higher morbidity and mortality long term.^39^

Similarly, markers of glucose intolerance and insulin resistance, including HbA_1c_ levels, fasting insulin level, HOMA-IR, and prevalence of prediabetes and type 2 diabetes, were significantly elevated in children and adolescents with obesity classes 4 to 5 (Figure 3), extending earlier results.^32,40,41^ The association between pediatric obesity and cardiometabolic risk factors is well established.^34,42^ This study found an even stronger correlation between extremely severe obesity and these conditions. The prevalence of pediatric metabolic syndrome was significantly higher among children with extremely severe obesity compared with those with obesity classes 1 to 3, alongside substantial increases in waist circumference and blood pressure and a decrease in HDL levels (Figure 4). These results underscore associations between extremely severe obesity and multiple cardiovascular health outcomes and the importance of comprehensive risk factor management.

The findings of this study with over 25 800 participants provide robust evidence supporting extremely severe obesity specifically as a public health emergency.^3^ The association with metabolic and cardiovascular complications necessitates urgent public health action, such as early prevention, targeted education, and the mobilization of resources.^43,44^ Moreover, policies aimed at reducing health disparities among different demographic groups are essential to address inequities. In light of the current national shortage of novel weight loss medications such as glucagon-like peptide-1 receptor agonists, fair and ethical allocation of those scarce medical resources should occur.^45^ The pediatric population with extremely severe obesity should be the preferred recipients of those medications (if lifestyle modifications do not lead to sufficient weight loss), as the medical intervention in this population would maximize benefits with lives, years of life, and healthy years of life saved compared with other populations. This medication allocation should occur irrespective of ethnicity, race, gender, or similar characteristics.

Future research should investigate the mechanisms linking extremely severe obesity to metabolic and cardiovascular risk factors and explore long-term health outcomes, including mortality. Longitudinal studies are needed to understand the long-term health trajectories in children with extremely severe obesity and to identify effective intervention strategies. In addition, randomized clinical trials should be designed specifically for children and adolescents with extremely severe obesity to evaluate novel therapeutics, as those individuals will likely benefit the most from effective weight loss medications.

### Strengths and Limitations

Strengths of this study include its use of nationally representative datasets with over 25 800 participants. To our knowledge, this study is the first to focus on extremely severe obesity (obesity classes 4 and 5), describe its increasing prevalence, and comprehensively detail its associated severe cardiometabolic comorbidities.

Limitations of the study include the absence of mortality data and reliance on noninvasive liver imaging rather than biopsies for the diagnosis of MASLD. However, a liver biopsy for each participant enrolled in NHANES would not be ethically defensible. Additionally, the accuracy and pediatric-specific cutoff values for noninvasive liver imaging are less established than those for adults, requiring cautious interpretation and further validation in children. Furthermore, the relatively small sample size of children and adolescents with extremely severe obesity in our study remains a limitation. As such, these findings should be confirmed in future studies.

## Conclusions

In this cohort study of US children and adolescents, prevalence of extremely severe obesity significantly increased over time, particularly among older adolescents and non-Hispanic Black participants. Extremely severe obesity was associated with severe metabolic and cardiovascular complications, including MASLD, prediabetes and diabetes, severe insulin resistance, and metabolic syndrome. The increasing prevalence of extremely severe pediatric obesity and its associated health consequences underscore the need for immediate public health interventions. By addressing this critical issue, we can improve the health of future generations and mitigate the long-term burden of obesity-related diseases.

## Notes

Supplement 1. **eMethods.** Supplementary Materials and Methods **eTable 1.** Overview of Proposed Definition of Pediatric Obesity Classes 4 and 5 in Context of Other Obesity Classes in Pediatric and Adult Populations **eTable 2.** Multiple Logistic Regression Using Demographic Parameters and Year of NHANES Dataset Iteration to Estimate Pediatric Extremely Severe Obesity in the Combined 2008-2023 NHANES Dataset **eTable 3.** Odds Ratio Per Multiple Logistic Regression Using Categorized Demographic Parameters and NHANES Dataset Iterations to Estimate Pediatric Extremely Severe Obesity in the Combined 2008-2023 NHANES Dataset With Over 25 800 Participants **eTable 4.** Odds Ratios of Metabolic Comorbidities Per Grouped Obesity Class **eFigure 1.** Prevalence of Pediatric Obesity Classes 4 to 5 in Adolescents and Non-Hispanic Black Individuals **eFigure 2.** Association of Extremely Severe Pediatric Obesity vs Obesity Class 3 With Metabolic Dysfunction–Associated Steatotic Liver Disease (MASLD) and More Advanced Liver Fibrosis **eFigure 3.** ALT and GGT Levels in Extremely Severe Pediatric Obesity **eFigure 4.** Association of Pediatric Obesity Classes 4 and 5 vs Class 3 With Prevalence of Prediabetes and Diabetes **eFigure 5.** Association of Pediatric Obesity Classes 4 and 5 vs Class 3 With Insulin Resistance **eFigure 6.** Prevalence of Metabolic Syndrome in Obesity Classes 3, 4, and 5 **eFigure 7.** Association of Pediatric Obesity Classes 4 and 5 With Cardiometabolic Risk Factors **eFigure 8.** Association of Obesity Classes With Metabolic Diseases, Stratified by Gender **eFigure 9.** Association of Obesity Classes With Metabolic Diseases, Stratified by Age **eFigure 10.** Association of Obesity Classes and Metabolic Diseases, Stratified by Ethnicity and Race
eReferences

Supplement 2.
Data Sharing Statement
