# Supplementary material for: Prevalence of Extremely Severe Obesity and Metabolic Dysfunction Among US Children and Adolescents
Source: JAMA Netw Open. 2025 Jul 16;8(7):e2521170. doi: 10.1001/jamanetworkopen.2025.21170 (PMC12268495; doi:10.1001/jamanetworkopen.2025.21170)
Supplement: Supplement 1. — eMethods. Supplementary Materials and Methods eTable 1. Overview of Proposed Definition of Pediatric Obesity Classes 4 and 5 in Context of Other Obesity Classes in Pediatric and Adult Populations eTable 2. Multiple Logistic Regression Using Demographic Parameters and Year of NHANES Dataset Iteration to Estimate Pediatric Extremely Severe Obesity in the Combined 2008-2023 NHANES Dataset eTable 3. Odds Ratio Per Multiple Logistic Regression Using Categorized Demographic Parameters and NHANES Dataset Iterations to Estimate Pediatric Extremely Severe Obesity in the Combined 2008-2023 NHANES Dataset With Over 25 800 Participants eTable 4. Odds Ratios of Metabolic Comorbidities Per Grouped Obesity Class eFigure 1. Prevalence of Pediatric Obesity Classes 4 to 5 in Adolescents and Non-Hispanic Black Individuals eFigure 2. Association of Extremely Severe Pediatric Obesity vs Obesity Class 3 With Metabolic Dysfunction–Associated Steatotic Liver Disease (MASLD) and More Advanced Liver Fibrosis eFigure 3. ALT and GGT Levels in Extremely Severe Pediatric Obesity eFigure 4. Association of Pediatric Obesity Classes 4 and 5 vs Class 3 With Prevalence of Prediabetes and Diabetes eFigure 5. Association of Pediatric Obesity Classes 4 and 5 vs Class 3 With Insulin Resistance eFigure 6. Prevalence of Metabolic Syndrome in Obesity Classes 3, 4, and 5 eFigure 7. Association of Pediatric Obesity Classes 4 and 5 With Cardiometabolic Risk Factors eFigure 8. Association of Obesity Classes With Metabolic Diseases, Stratified by Gender eFigure 9. Association of Obesity Classes With Metabolic Diseases, Stratified by Age eFigure 10. Association of Obesity Classes and Metabolic Diseases, Stratified by Ethnicity and Race eReferences [file jamanetwopen-e2521170-s001.pdf]

Supplementary Online Content

Münte E, Zhang X, Khurana A, Hartmann P. Prevalence of extremely severe obesity and metabolic dysfunction among US children and adolescents. *JAMA Netw Open*. 2025;8(7):e2521170. doi:10.1001/jamanetworkopen.2025.21170

eMethods. Supplementary Materials and Methods
eTable 1. Overview of Proposed Definition of Pediatric Obesity Classes 4 and 5 in Context of Other Obesity Classes in Pediatric and Adult Populations
eTable 2. Multiple Logistic Regression Using Demographic Parameters and Year of NHANES Dataset Iteration to Estimate Pediatric Extremely Severe Obesity in the Combined 2008-2023 NHANES Dataset
eTable 3. Odds Ratio Per Multiple Logistic Regression Using Categorized Demographic Parameters and NHANES Dataset Iterations to Estimate Pediatric Extremely Severe Obesity in the Combined 2008-2023 NHANES Dataset With Over 25 800 Participants
eTable 4. Odds Ratios of Metabolic Comorbidities Per Grouped Obesity Class
eFigure 1. Prevalence of Pediatric Obesity Classes 4 to 5 in Adolescents and Non-Hispanic Black Individuals
eFigure 2. Association of Extremely Severe Pediatric Obesity vs Obesity Class 3 With Metabolic Dysfunction–Associated Steatotic Liver Disease (MASLD) and More Advanced Liver Fibrosis
eFigure 3. ALT and GGT Levels in Extremely Severe Pediatric Obesity
eFigure 4. Association of Pediatric Obesity Classes 4 and 5 vs Class 3 With Prevalence of Prediabetes and Diabetes
eFigure 5. Association of Pediatric Obesity Classes 4 and 5 vs Class 3 With Insulin Resistance
eFigure 6. Prevalence of Metabolic Syndrome in Obesity Classes 3, 4, and 5
eFigure 7. Association of Pediatric Obesity Classes 4 and 5 With Cardiometabolic Risk Factors
eFigure 8. Association of Obesity Classes With Metabolic Diseases, Stratified by Gender
eFigure 9. Association of Obesity Classes With Metabolic Diseases, Stratified by Age
eFigure 10. Association of Obesity Classes and Metabolic Diseases, Stratified by Ethnicity and Race
eReferences

This supplementary material has been provided by the authors to give readers additional information about their work.

© 2025 Münte E et al. *JAMA Network Open*.

eMethods. Supplementary Materials and Methods

Demographic Variables

Race and ethnicity were self-reported by participants or their caregivers using standardized categories provided by the National Health and Nutrition Examination Survey (NHANES). The category “Other Hispanics” includes respondents who self-identify as Hispanic, but not as Mexican American. The category “Other Race” includes non-Hispanic individuals who identify with races other than White or Black, and multi-racial respondents, such as Asian American, Native Hawaiians, Pacific Islanders, American Indians, or Alaska Natives.

© 2025 Münte E et al. *JAMA Network Open*.

eTable 1. Overview of proposed definition of pediatric obesity classes 4 and 5 (extremely severe obesity) in context of other obesity classes in pediatric and adult populations.

Obesity Class	Pediatric	Adult[2]
**1**	BMI ≥ 95th to <120% of 95th percentile for age and gender	BMI ≥ 30
**2**	BMI ≥ 120% to <140% of 95th percentile for age and gender	BMI ≥ 35
**3**	BMI ≥ 140% to <160% of 95th percentile for age and gender	BMI ≥ 40
**4**	BMI ≥ 160% to <180% of 95th percentile for age and gender	BMI ≥ 50
**5**	BMI ≥ 180% of 95th percentile for age and gender	BMI ≥ 60

BMI, Body Mass Index.

© 2025 Münte E et al. *JAMA Network Open*.

eTable 2. Multiple logistic regression using demographic parameters and year of NHANES dataset iteration to estimate pediatric extremely severe obesity in the combined NHANES dataset 2008-2023 with over 25,800 participants.

Variable	Estimate	95% CI	*p* value
**Mexican American**	**0.77**	**0.24 - 1.32**	**0.004**
**Non-Hispanic Black**	**1.45**	**1.00 - 1.93**	**<0.001**
Other Hispanic	0.25	-0.51 - 0.94	0.500
Other Race - Including Multi-Racial	0.34	-0.29 - 0.95	0.282
**Male Gender**	**0.4**	**0.09 - 0.73**	**0.013**
**Age [years]**	**0.15**	**0.12 - 0.19**	**<0.001**
**NHANES Dataset [every 2-3 years]**	**0.08**	**0.04 - 0.11**	**<0.001**

CI, Confidence interval. Respective race/ethnicity was compared with non-Hispanic Whites. “Other Hispanics” includes respondents who self-identify as Hispanic, but not as Mexican American. “Other Race” includes non-Hispanic individuals who identify with races other than White or Black, and multi-racial respondents. Bold font indicates statistical significance ($p<0.05$).

© 2025 Münte E et al. *JAMA Network Open*.

eTable 3. Odds ratio per multiple logistic regression using categorized demographic parameters and NHANES dataset iterations to estimate pediatric extremely severe obesity in the combined NHANES dataset 2008-2023 with over 25,800 participants.

Variable	Odds Ratio	95% CI	*p* value
**Non-Hispanic Black vs Other Races/Ethnicities**	**2.89**	**2.11 - 3.96**	**<0.001**
**Male vs Female**	**1.50**	**1.09 - 2.07**	**0.013**
**Age ≥ 12 years vs < 12 years**	**3.19**	**2.29 - 4.52**	**<0.001**
**Combined 2020 and 2023 NHANES vs Other Datasets**	**1.97**	**1.43 - 2.70**	**<0.001**

CI, Confidence interval. Bold font indicates statistical significance ($p<0.05$).

© 2025 Münte E et al. *JAMA Network Open*.

eTable 4. Odds ratio of metabolic comorbidities per grouped obesity classes.

	No Obesity	Class 1-3	Class 4-5
**MASLD**	0.04 [0.03;0.05]	Reference	6.74 [3.30;15.7]
**Hepatic Steatosis Grade 1+**	0.06 [0.04;0.07]	Reference	6.74 [3.30;15.7]
**Hepatic Steatosis Grade 2+**	0.06 [0.04;0.07]	Reference	3.31 [1.87;6.00]
**Hepatic Steatosis Grade 3**	0.03 [0.01;0.07]	Reference	5.09 [2.57;9.66]
**Hepatic Fibrosis Stage 2+**	0.41 [0.31;0.55]	Reference	5.01 [2.68;9.15]
**Hepatic Fibrosis Stage 3+**	0.53 [0.32;0.90]	Reference	10.00 [4.51;21.2]
**Prediabetes/Diabetes**	0.36 [0.32;0.41]	Reference	4.94 [3.41;7.14]
**Severe Insulin Resistance**	0.08 [0.05;0.14]	Reference	8.05 [3.70;17.0]
**Metabolic Syndrome**	0.08 [0.07;0.09]	Reference	1.99 [1.45;2.73]

Odds ratios and in brackets 95% confidence intervals are shown with the group without obesity serving as the reference group. MASLD, Metabolic Dysfunction-Associated Steatotic Liver Disease.

© 2025 Münte E et al. *JAMA Network Open*.

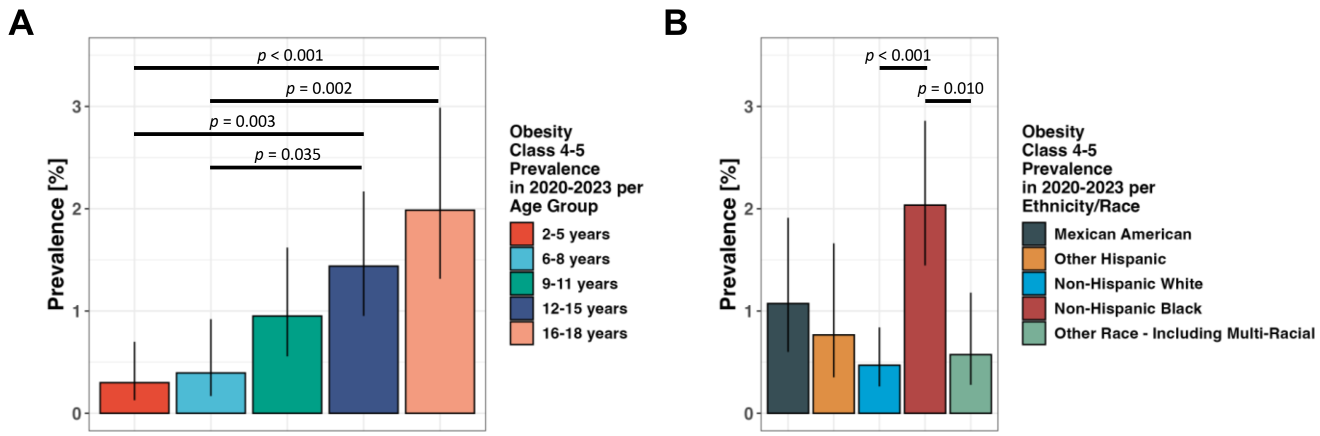

eFigure 1. The prevalence of pediatric obesity classes 4-5 in adolescents and non-Hispanic Black individuals. (A) Relative prevalence of obesity classes 4-5 per age group in 2020-2023 (total, n=6,940; 2-5 years, n=1,669; 6-8 years, n=1,267; 9-11 years, n=1,367; 12-15 years, n=1,529; 16-18 years, n=1,108). (B) Relative prevalence of obesity classes 4-5 per ethnicity/race in 2020-2023 (total, n=6.940; Mexican American, n=1,025; Other Hispanic, n=783; Non-Hispanic White, n=2,340; Non-Hispanic Black, n=1,572; Other Race - Including Multi-Racial, n=1,220). Adjusted *p* values after Holm correction are shown. Statistical significance is indicated by $p<0.05$.

© 2025 Münte E et al. *JAMA Network Open*.

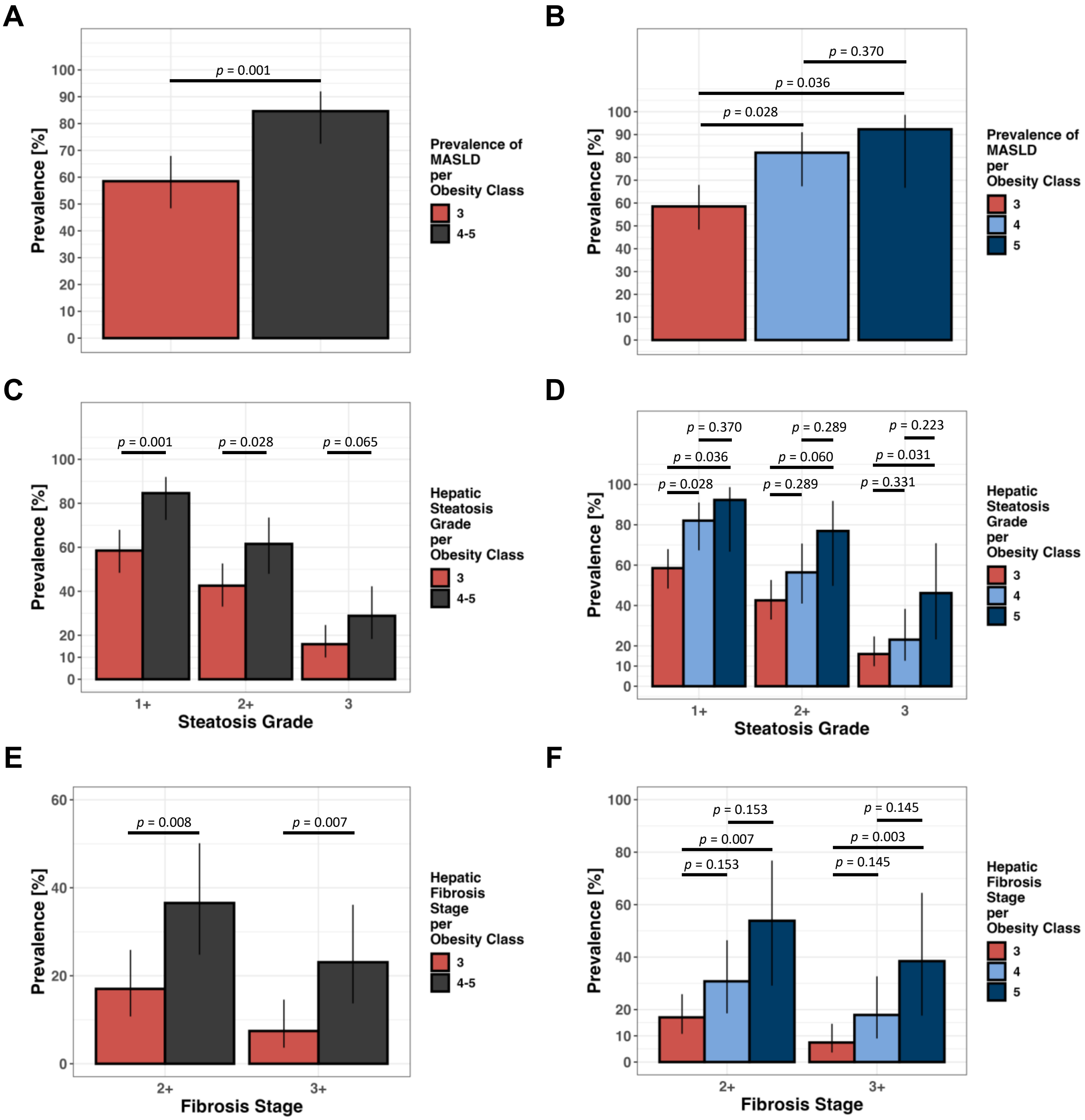

eFigure 2. Extremely severe pediatric obesity is associated with metabolic dysfunction-associated steatotic liver disease (MASLD) and more advanced liver fibrosis compared with obesity class 3. (A-B) Relative prevalence of pediatric MASLD per obesity class (n=146). (C-D) Relative prevalence of hepatic steatosis grade 1+, 2+,

© 2025 Münte E et al. *JAMA Network Open*.

and 3+ per obesity class (n=146). (E-F) Relative prevalence of hepatic fibrosis stage 2+ and 3+ per obesity class (n=146). Adjusted *p* values after Holm correction are shown. 95% confidence intervals are indicated. Statistical significance is indicated by $p<0.05$. CAP, Controlled Attenuation Parameter; MASLD, Metabolic Dysfunction-Associated Steatotic Liver Disease.

© 2025 Münte E et al. *JAMA Network Open*.

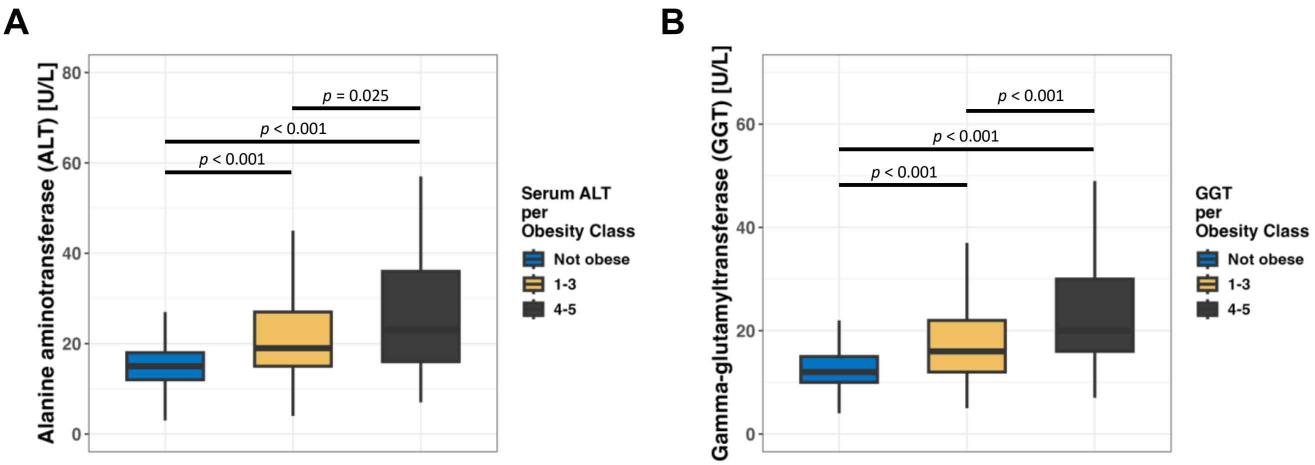

eFigure 3. ALT and GGT levels are high in extremely severe pediatric obesity. (A) Alanine aminotransferase (ALT) per obesity class (n=7,185). (B) Gamma-glutamyltransferase (GGT) per obesity class (n=7,185). Adjusted *p* values after Holm correction are shown. For the box and whisker plots, the box extends from the 25th to 75th percentile, with the center line indicating the median; the bottom whiskers indicate the minimum value of the data that is within 1.5 times the interquartile range under the 25th percentile, and the top whiskers indicate the maximum value of the data that is within 1.5 times the interquartile range over the 75th percentile. Statistical significance is indicated by $p<0.05$. ALT, alanine aminotransferase; GGT, Gamma-glutamyltransferase.

© 2025 Münte E et al. *JAMA Network Open*.

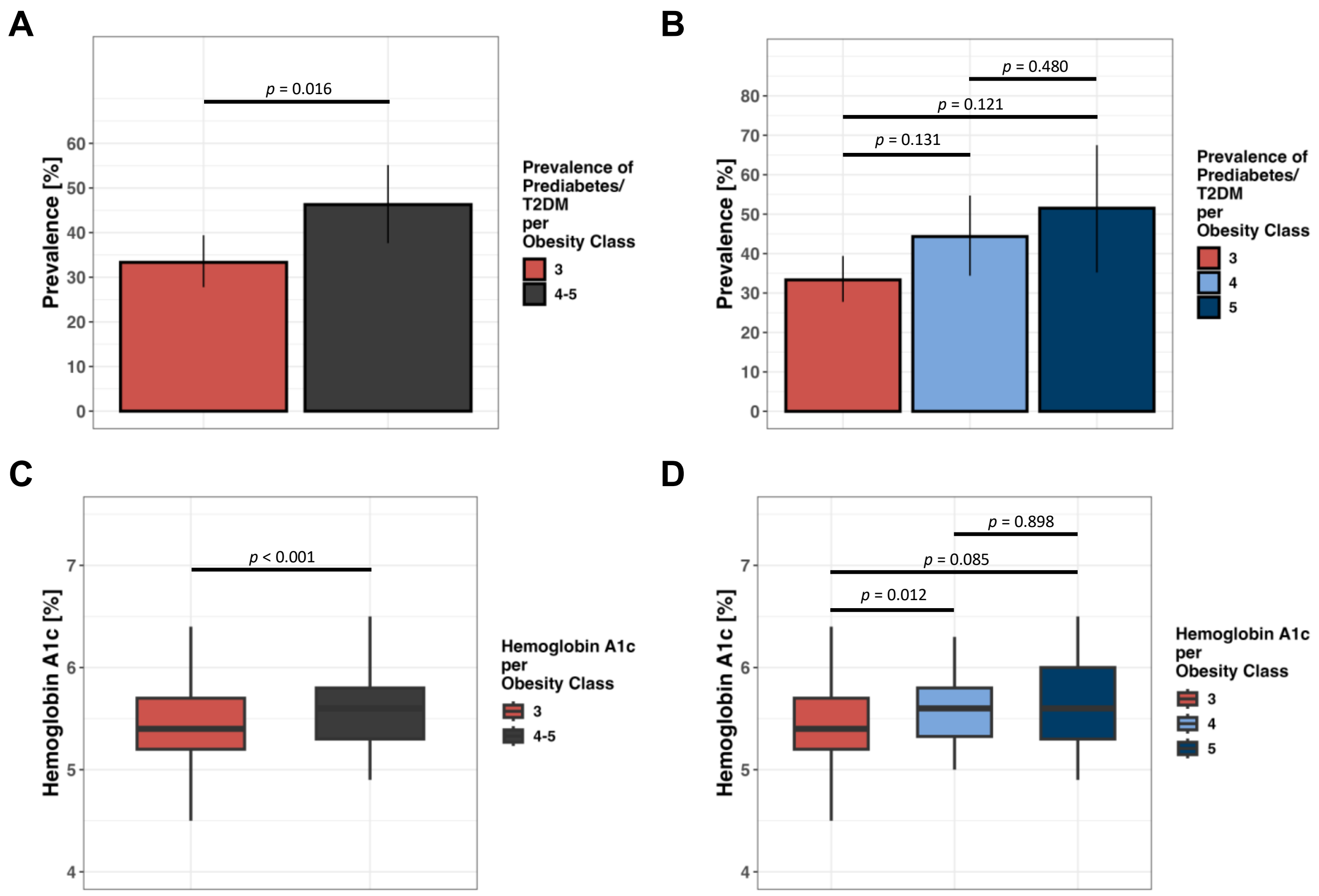

eFigure 4. Pediatric obesity classes 4 and 5 are associated with more prediabetes/diabetes compared with class 3. (A-B) Relative prevalence of prediabetes/T2DM per obesity class (n=370). (C-D) Hemoglobin A1c per obesity class (n=256). Adjusted *p* values after Holm correction are shown. For the bar plots (A-B), 95% confidence intervals are indicated. For the box and whisker plots (C-D), the box extends from the 25th to 75th percentile, with the center line indicating the median; the bottom whiskers indicate the minimum value of the data that is within 1.5 times the interquartile range under the 25th percentile, and the top whiskers indicate the maximum value of the data that is within 1.5 times the interquartile range over the 75th percentile. Statistical significance is indicated by $p<0.05$. T2DM, Type 2 Diabetes Mellitus.

© 2025 Münte E et al. *JAMA Network Open*.

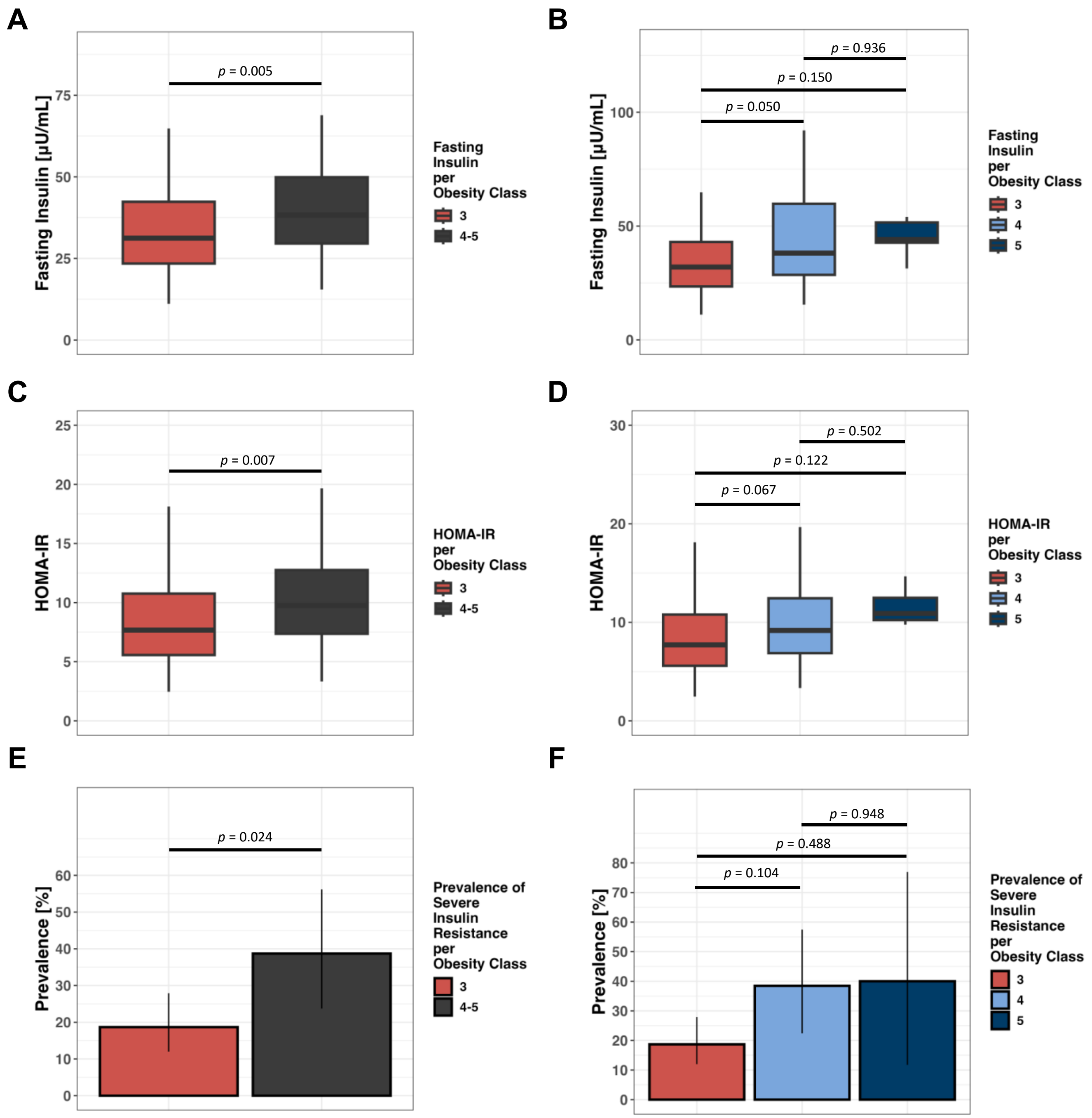

eFigure 5. Pediatric obesity classes 4 and 5 are associated with insulin resistance compared with class 3. (A-B) Fasting insulin per obesity class (n=122). (C-D) HOMA-IR per obesity class (n=121). (E-F) Prevalence of severe insulin resistance per obesity class (n=122). Adjusted *p* values after Holm correction are shown. For the box and whisker

© 2025 Münte E et al. *JAMA Network Open*.

plots (A-D), the box extends from the 25th to 75th percentile, with the center line indicating the median; the bottom whiskers indicate the minimum value of the data that is within 1.5 times the interquartile range under the 25th percentile, and the top whiskers indicate the maximum value of the data that is within 1.5 times the interquartile range over the 75th percentile. For the bar plots (E-F), 95% confidence intervals are indicated. Statistical significance is indicated by $p<0.05$. HOMA-IR, Homeostatic Model Assessment of Insulin Resistance; T2DM, Type 2 Diabetes Mellitus.

© 2025 Münte E et al. *JAMA Network Open*.

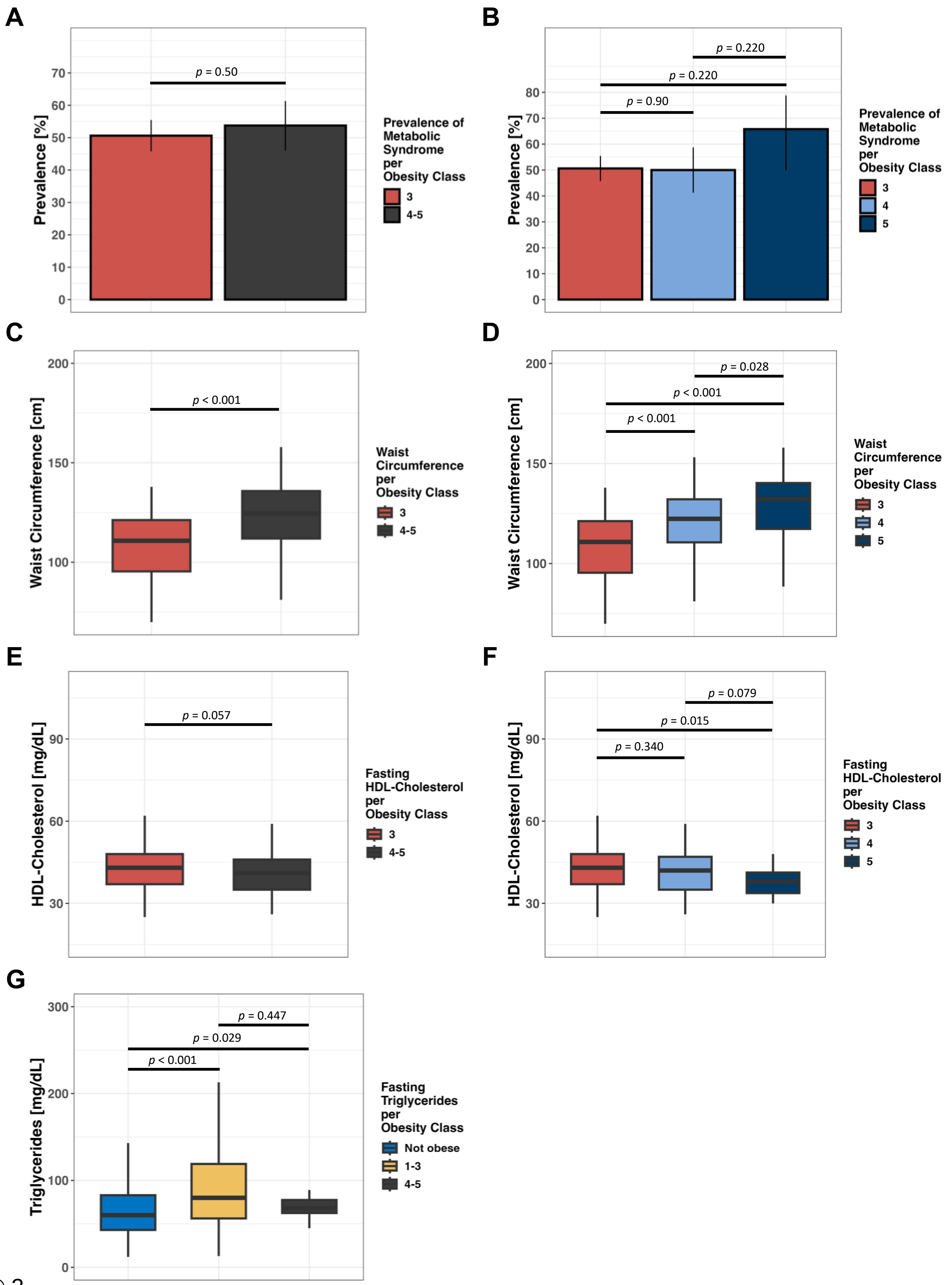

© 20[illegible] Munte E et al. JAMA Network Open.

eFigure 6. Metabolic syndrome prevalence in classes 3, 4, and 5 obesity. (A-B) Relative prevalence of metabolic syndrome per obesity class after multiple imputation of missing cardiometabolic parameters (n=565). (C-D) Waist circumference per obesity class (n=486). (E-F) HDL-cholesterol per obesity class (n=392). (G) Triglycerides per obesity class (n=3,348). Adjusted *p* values after Holm correction are shown. For the bar plots (A-B), 95% confidence intervals are indicated. For the box and whisker plots (C-G), the box extends from the 25th to 75th percentile, with the center line indicating the median; the bottom whiskers indicate the minimum value of the data that is within 1.5 times the interquartile range under the 25th percentile, and the top whiskers indicate the maximum value of the data that is within 1.5 times the interquartile range over the 75th percentile. Statistical significance is indicated by $p<0.05$. HDL, High-Density Lipoprotein.

© 2025 Münte E et al. *JAMA Network Open*.

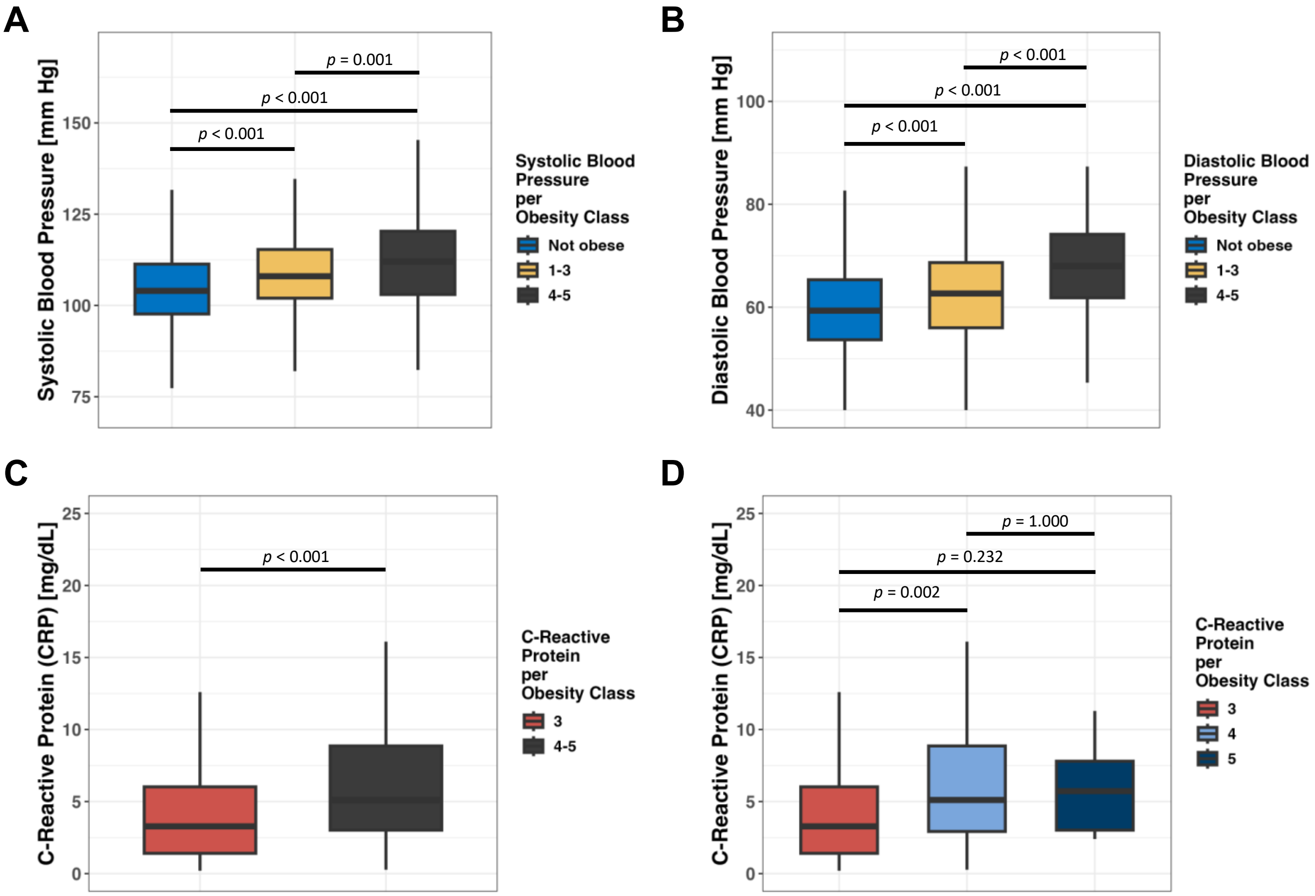

eFigure 7. Pediatric obesity classes 4 and 5 are associated with cardiometabolic risk factors. (A) Systolic blood pressure per obesity class (n=13,935). (B) Diastolic blood pressure per obesity class (n=13,935). (C-D) C-Reactive Protein per obesity class (n=254). Adjusted *p* values after Holm correction are shown. For the box and whisker plots, the box extends from the 25th to 75th percentile, with the center line indicating the median; the bottom whiskers indicate the minimum value of the data that is within 1.5 times the interquartile range under the 25th percentile, and the top whiskers indicate the maximum value of the data that is within 1.5 times the interquartile range over the 75th percentile. Statistical significance is indicated by *p*<0.05.

© 2025 Münte E et al. *JAMA Network Open*.

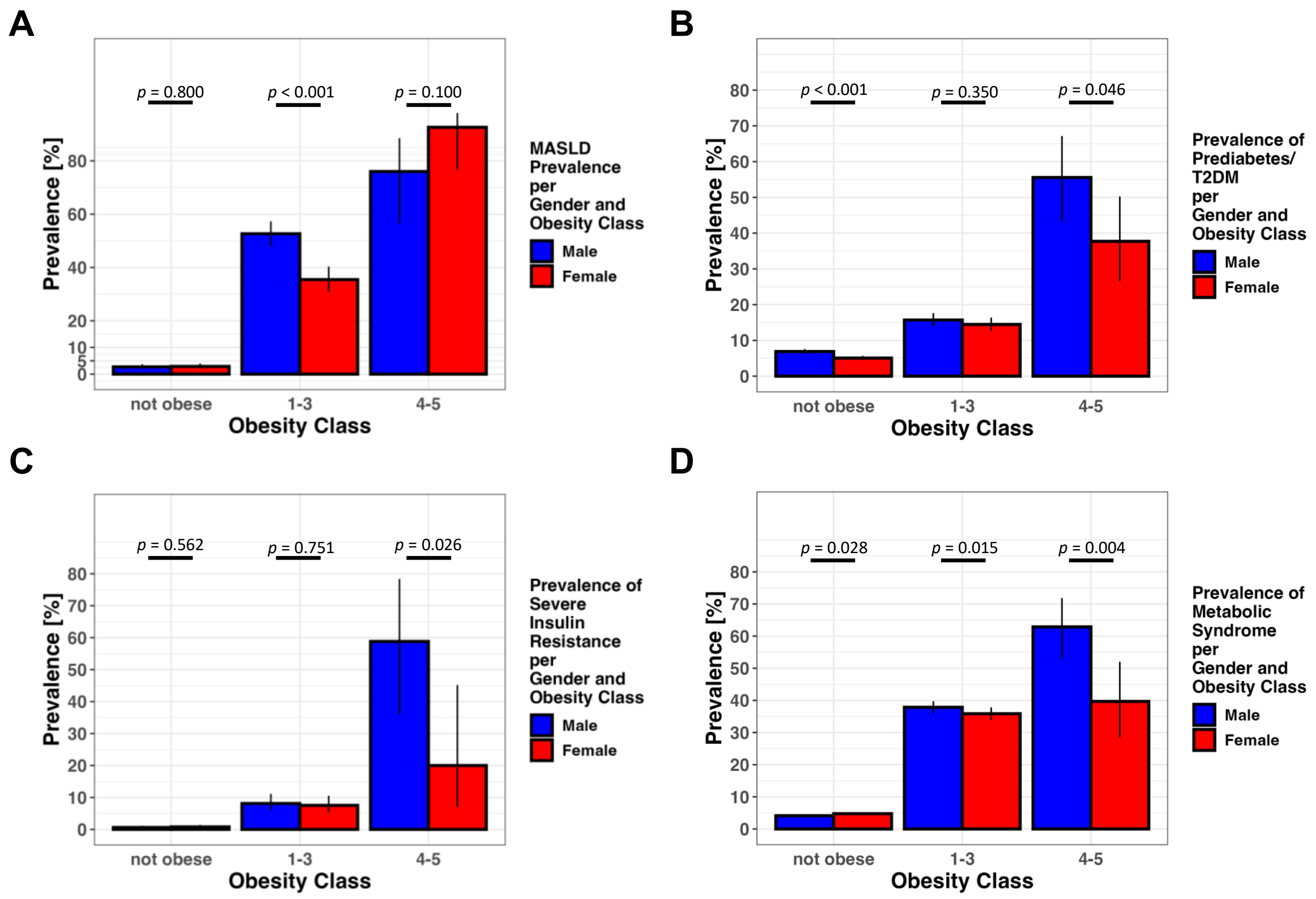

eFigure 8. Association of obesity classes and metabolic diseases, stratified by gender. (A) Relative prevalence of pediatric MASLD per obesity class and gender (n=3,483). (B) Relative prevalence of prediabetes/T2DM per obesity class and gender (n=13,690). (C) Relative prevalence of severe insulin resistance per obesity class and gender (n=3,716). (D) Relative prevalence of metabolic syndrome per obesity class and gender after multiple imputation of missing cardiometabolic parameters (n=25,847). Adjusted *p* values after Holm correction are shown. For the bar plots, 95% confidence intervals are indicated. Statistical significance is indicated by $p<0.05$. MASLD, Metabolic Dysfunction-Associated Steatotic Liver Disease; T2DM, Type 2 Diabetes Mellitus.

© 2025 Münte E et al. *JAMA Network Open*.

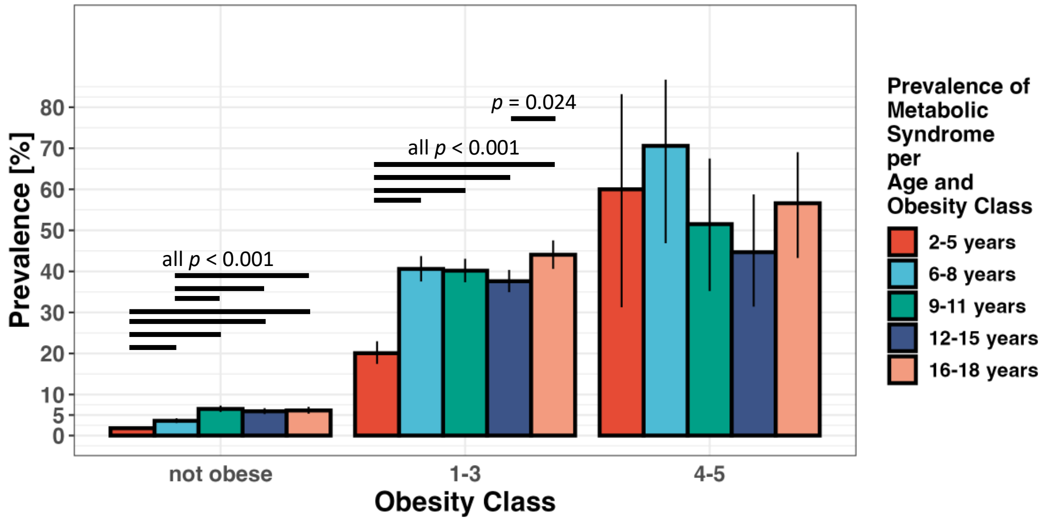

eFigure 9. Association of obesity classes and metabolic diseases, stratified by age. Relative prevalence of metabolic syndrome per obesity class and age after multiple imputation of missing cardiometabolic parameters (n=25,847). Adjusted *p* values after Holm correction are shown. For the bar plots, 95% confidence intervals are indicated. Statistical significance is indicated by $p<0.05$. Only significant p values are shown.

© 2025 Münte E et al. *JAMA Network Open*.

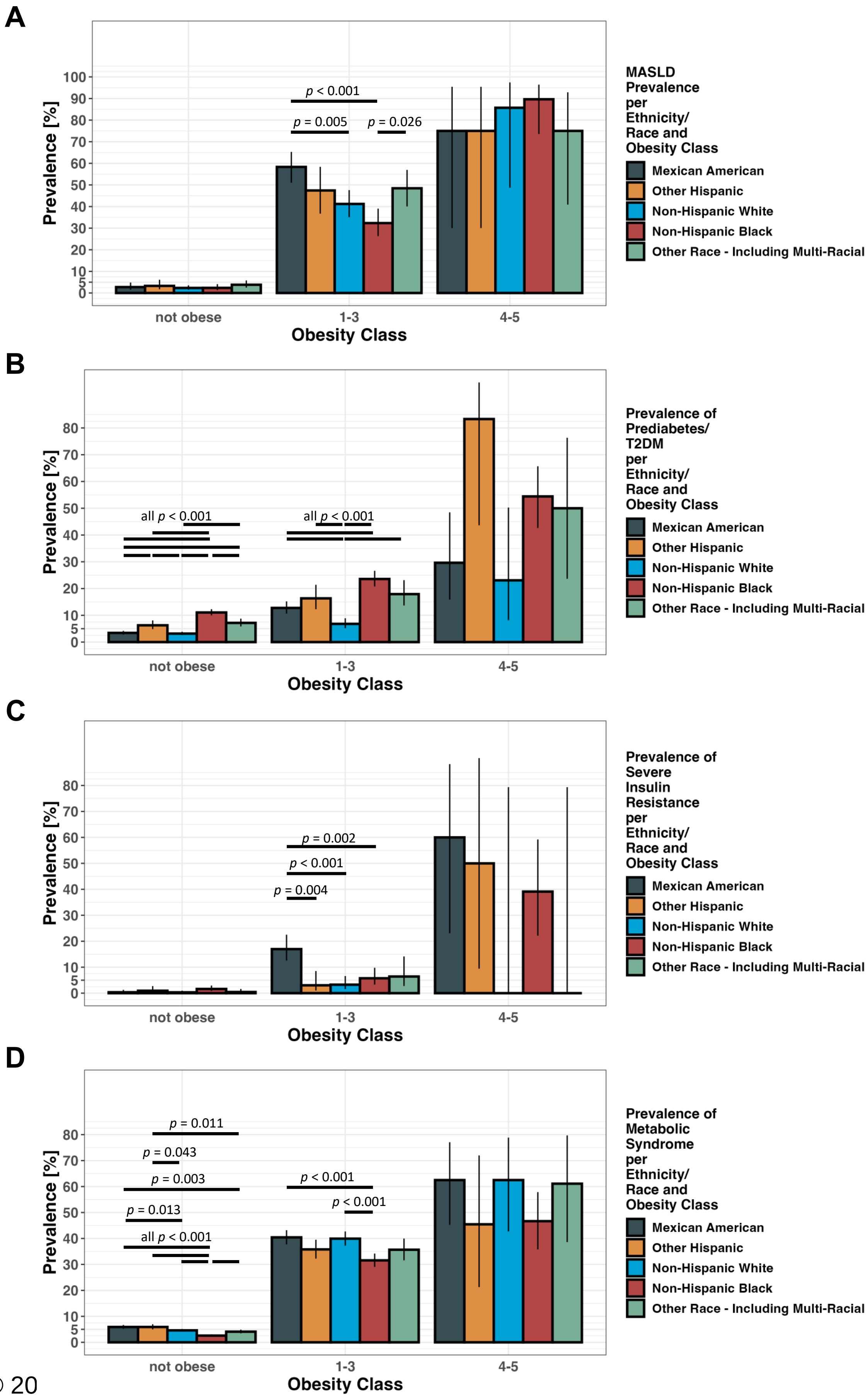
A
Prevalence [%]
p < 0.001
p = 0.005
p = 0.026
not obese
1-3
4-5
Obesity Class
MASLD Prevalence per Ethnicity/ Race and Obesity Class
Mexican American
Other Hispanic
Non-Hispanic White
Non-Hispanic Black
Other Race - Including Multi-Racial
B
Prevalence [%]
all p < 0.001
all p < 0.001
not obese
1-3
4-5
Obesity Class
Prevalence of Prediabetes/ T2DM per Ethnicity/ Race and Obesity Class
Mexican American
Other Hispanic
Non-Hispanic White
Non-Hispanic Black
Other Race - Including Multi-Racial
C
Prevalence [%]
p = 0.002
p < 0.001
p = 0.004
not obese
1-3
4-5
Obesity Class
Prevalence of Severe Insulin Resistance per Ethnicity/ Race and Obesity Class
Mexican American
Other Hispanic
Non-Hispanic White
Non-Hispanic Black
Other Race - Including Multi-Racial
D
Prevalence [%]
p = 0.011
p = 0.043
p = 0.003
p = 0.013
all p < 0.001
p < 0.001
p < 0.001
not obese
1-3
4-5
Obesity Class
Prevalence of Metabolic Syndrome per Ethnicity/ Race and Obesity Class
Mexican American
Other Hispanic
Non-Hispanic White
Non-Hispanic Black
Other Race - Including Multi-Racial

© 20

eFigure 10. Association of obesity classes and metabolic diseases, stratified by ethnicity/race. (A) Relative prevalence of pediatric MASLD per obesity class and ethnicity/race (n=3,483). (B) Relative prevalence of prediabetes/T2DM per obesity class and ethnicity/race (n=13,690). (C) Relative prevalence of severe insulin resistance per obesity class and ethnicity/race (n=3,716). (D) Relative prevalence of metabolic syndrome per obesity class and ethnicity/race after multiple imputation of missing cardiometabolic parameters (n=25,847). Adjusted *p* values after Holm correction are shown. For the bar plots, 95% confidence intervals are indicated. Statistical significance is indicated by $p<0.05$. Only significant p values are shown. MASLD, Metabolic Dysfunction-Associated Steatotic Liver Disease; T2DM, Type 2 Diabetes Mellitus.

© 2025 Münte E et al. *JAMA Network Open*.

eReferences

1. Skinner AC, Perrin EM, Moss LA, Skelton JA. Cardiometabolic Risks and Severity of Obesity in Children and Young Adults. *N Engl J Med*. Oct 2015;373(14):1307-17. doi:10.1056/NEJMoa1502821
2. Poirier P, Cornier MA, Mazzone T, et al. Bariatric surgery and cardiovascular risk factors: a scientific statement from the American Heart Association. *Circulation*. Apr 19 2011;123(15):1683-701. doi:10.1161/CIR.0b013e3182149099
3. Centers for Disease Control and Prevention (CDC). Percentile data files with LMS values. Accessed 08/03/2024, http://www.cdc.gov/growthcharts/percentile_data_files.htm, accessed on 8/4/24
4. Rinella ME, Lazarus JV, Ratziu V, et al. A multisociety Delphi consensus statement on new fatty liver disease nomenclature. *Hepatology*. Dec 01 2023;78(6):1966-1986. doi:10.1097/HEP.0000000000000520
5. Sharma AK, Metzger DL, Daymont C, Hadjiyannakis S, Rodd CJ. LMS tables for waist-circumference and waist-height ratio Z-scores in children aged 5-19 y in NHANES III: association with cardio-metabolic risks. *Pediatr Res*. Dec 2015;78(6):723-9. doi:10.1038/pr.2015.160
6. Martin B, DeWitt PE, Albers D, Bennett TD. Development of a Pediatric Blood Pressure Percentile Tool for Clinical Decision Support. *JAMA Netw Open*. Oct 03 2022;5(10):e2236918. doi:10.1001/jamanetworkopen.2022.36918
7. Runge JH, van Giessen J, Draijer LG, et al. Accuracy of controlled attenuation parameter compared with ultrasound for detecting hepatic steatosis in children with severe obesity. *Eur Radiol*. Mar 2021;31(3):1588-1596. doi:10.1007/s00330-020-07245-2
8. Nobili V, Vizzutti F, Arena U, et al. Accuracy and reproducibility of transient elastography for the diagnosis of fibrosis in pediatric nonalcoholic steatohepatitis. *Hepatology*. Aug 2008;48(2):442-8. doi:10.1002/hep.22376
9. de Ferranti SD, Gauvreau K, Ludwig DS, Neufeld EJ, Newburger JW, Rifai N. Prevalence of the metabolic syndrome in American adolescents: findings from the Third National Health and Nutrition Examination Survey. *Circulation*. Oct 19 2004;110(16):2494-7. doi:10.1161/01.CIR.0000145117.40114.C7
10. Magge SN, Goodman E, Armstrong SC, NUTRITION CO, ENDOCRINOLOGY SO, OBESITY SO. The Metabolic Syndrome in Children and Adolescents: Shifting the Focus to Cardiometabolic Risk Factor Clustering. *Pediatrics*. Aug 2017;140(2)doi:10.1542/peds.2017-1603
11. van Buuren S, Groothuis-Oudshoorn K. mice: Multivariate Imputation by Chained Equations in R. *Journal of Statistical Software*. 2011;45(3):1-67. doi:10.18637/jss.v045.i03
12. Breiman L. *Classification and regression trees*. Routledge; 2017.
13. Rubin DB. *Multiple imputation for nonresponse in surveys*. vol 81. John Wiley & Sons; 2004.
14. Matthews DR, Hosker JP, Rudenski AS, Naylor BA, Treacher DF, Turner RC. Homeostasis model assessment: insulin resistance and beta-cell function from fasting plasma glucose and insulin concentrations in man. *Diabetologia*. Jul 1985;28(7):412-9. doi:10.1007/BF00280883
15. Keskin M, Kurtoglu S, Kendirci M, Atabek ME, Yazici C. Homeostasis model assessment is more reliable than the fasting glucose/insulin ratio and quantitative insulin sensitivity check index

© 2025 Münte E et al. *JAMA Network Open*.

for assessing insulin resistance among obese children and adolescents. *Pediatrics*. Apr 2005;115(4):e500-3. doi:10.1542/peds.2004-1921
16. Tritos NA, Mantzoros CS. Clinical review 97: Syndromes of severe insulin resistance. *J Clin Endocrinol Metab*. Sep 1998;83(9):3025-30. doi:10.1210/jcem.83.9.5143

© 2025 Münte E et al. *JAMA Network Open*.
