# Supplementary material for: Prevalence of Extremely Severe Obesity and Metabolic Dysfunction Among US Children and Adolescents
Source: JAMA Netw Open. 2025 Jul 16;8(7):e2521170. doi: 10.1001/jamanetworkopen.2025.21170 (PMC12268495; doi:10.1001/jamanetworkopen.2025.21170)
Supplement: Supplement 2. — Data Sharing Statement [file jamanetwopen-e2521170-s002.pdf]

Data Sharing Statement

Münte. Prevalence of Extremely Severe Obesity and Metabolic Dysfunction Among US Children and Adolescents. *JAMA Netw Open*. Published July 16, 2025.
doi:10.1001/jamanetworkopen.2025.21170

Data
Data available: No

Additional Information
Explanation for why data not available: NHANES datasets are publicly available
